# Trifluridine/Tipiracil and Oxaliplatin as Induction Chemotherapy in Resectable Esophageal and Gastroesophageal Junction Adenocarcinoma: A Phase II Study

**DOI:** 10.1002/cam4.70835

**Published:** 2025-04-08

**Authors:** Sarbajit Mukherjee, Yu Fujiwara, Christos Fountzilas, Harsha Pattnaik, Sarah Chatley, Deepak Vadehra, Moshim Kukar, Kristopher Attwood, Anthony George, Shailesh Advani, Han Yu, Kayla Catalfamo, Alyson Brown, Erik Spickard, Arkarachai Fungtammasan, Sagila George, Chih‐Yi Liao, Renuka Iyer, Hassan Hatoum

**Affiliations:** ^1^ Roswell Park Comprehensive Cancer Center Buffalo New York USA; ^2^ Miami Cancer Institute, Baptist Health South Florida Miami Florida USA; ^3^ Natera, Inc. Austin Texas USA; ^4^ Stephenson Cancer Center – University of Oklahoma Health Sciences Center Oklahoma City Oklahoma USA; ^5^ The University of Chicago Medical Center & Biological Sciences Chicago Illinois USA

**Keywords:** circulating tumor DNA, esophageal cancer, induction chemotherapy, pathologic complete response, trifluridine/tipiracil

## Abstract

**Background:**

Preoperative chemoradiation (CRT) followed by surgery for localized esophageal and gastroesophageal junction adenocarcinoma (EGAC) is a standard of care with a pathologic complete response (pCR) rate of 20%. We evaluated a novel combination of trifluridine/tipiracil with oxaliplatin as induction chemotherapy (IC) followed by CRT.

**Methods:**

We enrolled patients with potentially resectable localized EGAC (T3, T4aN0, or node‐positive disease) in this open‐label, single‐arm, multicenter, Phase II trial between January 2020 and October 2022. Patients received three cycles of IC with trifluridine/tipiracil and oxaliplatin and then underwent concurrent CRT with weekly carboplatin and paclitaxel followed by surgery. The primary objective was to evaluate the pCR rate. The secondary objectives were to evaluate 2‐year progression‐free survival (PFS), 2‐year overall survival (OS), and toxicities. Circulating tumor DNA (ctDNA) was measured at prespecified intervals to assess its correlation with clinical outcomes.

**Results:**

Of the 22 enrolled patients, 19 (86.4%) were male and 20 (90.9%) were Caucasian. The median age was 61 years, and 12 (54.5%) had their primary disease at the gastroesophageal junction. Twenty (90.9%) patients had T3 disease, and 15 (68.2%) had node‐positive disease. Only two patients had pCRs, and an additional five had near pCRs. Since we could not meet our predefined pCR rate at the interim analysis, the study was closed. After a median follow‐up of 15.8 months, 2‐year OS and PFS were 43% and 41%, respectively. ctDNA clearance was associated with a significantly higher OS rate (*p* = 0.012) and PFS rate (*p* = 0.008). Nausea (59.1%) and fatigue (59.1%) were common treatment‐related adverse events (AEs); nine (40.9%) patients had Grade 3 or higher AEs.

**Conclusion:**

IC with trifluridine/tipiracil and oxaliplatin followed by CRT did not improve pCR rate in resectable EGAC compared to pCR from previous reports with CRT alone. We found a correlation between ctDNA clearance and improved survival, which merits further investigation.

**Clinical Trial Information:**

NCT04097028.

## 1 Introduction

Esophageal cancer is a highly lethal cancer, and the incidence of esophageal adenocarcinoma has increased in Western countries in recent decades [1]. According to the American Cancer Society, there will be 22,370 new esophageal cancer cases, leading to 16,130 deaths in 2024 in the United States [2]. The incidence of gastroesophageal junction (GEJ) adenocarcinoma has also increased steadily in the United States since the 1970s [3]. Esophageal and GEJ adenocarcinoma (EGAC) present as a locoregional disease in approximately 32% of the cases, with 5‐year survival rarely exceeding 30% [4].

For more than a decade, neoadjuvant chemoradiation (CRT) followed by surgery has been a standard of care in EGAC based on the CROSS trial [5]. However, long‐term follow‐up data from the CROSS trial showed that CRT followed by surgery is ineffective in preventing distant metastasis compared to surgery alone, although CRT therapy significantly reduced the likelihood of locoregional recurrence [6]. Regarding the utility of neoadjuvant chemotherapy, the Neo‐AEGIS study randomized EGAC patients to perioperative chemotherapy versus CRT, demonstrating no significant difference in overall survival (OS) but an improved pathologic complete response (pCR), major pathological response, and R0 resection rates in CRT therapy [7].

Induction chemotherapy (IC) has been investigated in several clinical trials to augment the efficacy of preoperative CRT. A Phase II study conducted between 2005 and 2011, which randomized EGAC patients to CRT with or without IC with modified oxaliplatin, leucovorin, and fluorouracil (FOLFOX), demonstrated a nonsignificant increase in pCR rate with IC though no survival benefit was observed [8]. The CALGB 80803 study took a positron emission tomography (PET)‐response‐guided approach to tailor the chemotherapy backbone in resectable EGAC [9]. In this trial, patients were randomized to FOLFOX or carboplatin plus paclitaxel as IC, and patients with a PET response (≥ 35% decrease in SUV) continued the same chemotherapy backbone during CRT. In contrast, PET non‐responders (< 35% decrease in SUV) switched over to the alternative chemotherapy regimen during CRT. The PET‐guided approach was effective in improving the pCR rate among non‐responders, suggesting a possible role for IC in resectable EGAC, particularly those IC regimens with novel combinations.

Trifluridine/tipiracil has a distinct mechanism of action, differing from that of 5‐fluorouracil (5‐FU). 5‐FU leads to cytotoxicity by inhibiting thymidylate synthase (TS) without being directly incorporated into the DNA. Trifluridine, in addition to inhibiting TS, is incorporated into the DNA, causing single‐ and double‐stranded DNA breaks and DNA instability [10]. Tipiracil inhibits thymidine phosphorylase, an enzyme responsible for the metabolism of trifluridine, and also has potential antiangiogenic properties of which the clinical significance has yet to be established [11]. Inclusion of tipiracil increases trifluridine exposure by inhibiting its metabolism by thymidine phosphorylase. A randomized Phase III study evaluated the efficacy of trifluridine/tipiracil in refractory advanced gastric or GEJ cancer, showing that trifluridine/tipiracil prolonged progression‐free survival (PFS) and OS in a heavily pretreated population [12].

Recent studies have highlighted the potential of circulating tumor DNA (ctDNA) in assessing molecular residual disease in upper gastrointestinal cancers [13, 14, 15]. ctDNA has emerged as a promising minimally invasive biomarker for early detection of cancer relapse, risk stratification, and monitoring treatment response [16]. Although ctDNA shows promise in colorectal cancer management, evidence for upper gastrointestinal tumors remains limited [17]. Thus, prospective, multicenter, and interventional studies are needed to establish the clinical utility of ctDNA in guiding patient management and improving outcomes in upper gastrointestinal cancers [18].

In this study, we hypothesized that IC with a novel combination of trifluridine/tipiracil and oxaliplatin before standard preoperative CRT will increase the pCR rate in localized EGAC. Therefore, we performed a Phase II study to evaluate the pCR rate, survival outcomes, and toxicity from this novel IC strategy. We also examined the utility of monitoring ctDNA by correlating it to metabolic response and survival.

## 2 Materials and Methods

### 2.1 Design

**FIGURE 1:**
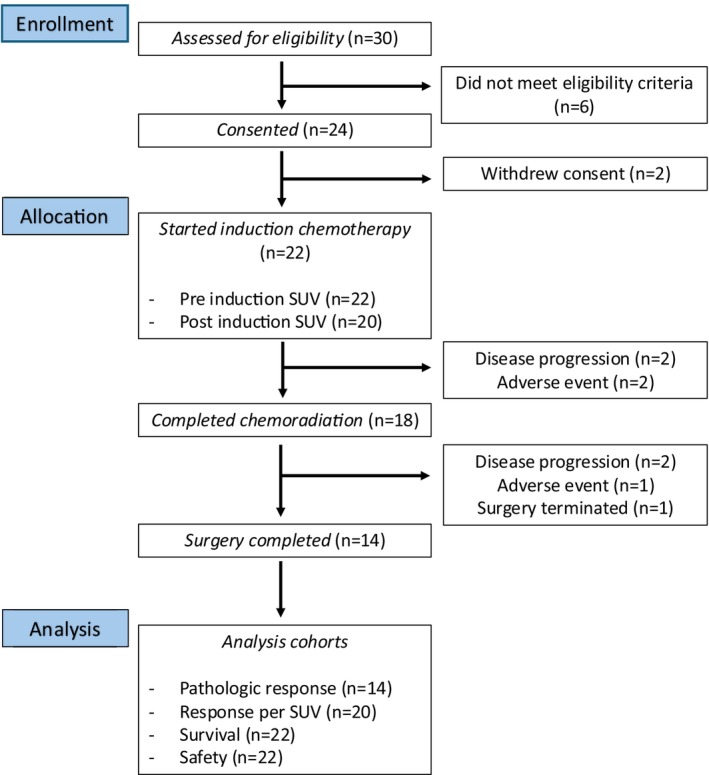
Consort diagram of this study. This figure illustrates a consort diagram of this study from patient enrollment, allocation, and analysis. SUV, standardized uptake value.

This was an open‐label, multicenter, Phase II study evaluating the efficacy of trifluridine/tipiracil administered with oxaliplatin before CRT in participants with adenocarcinoma of the esophagus or GE junction (Figure 1). The study was conducted according to the Declaration of Helsinki principles and approved by the institutional review boards of all participating institutions. The study was registered at ClinicalTrials.gov (NCT04097028).

### 2.2 Objectives

The primary objective of this study was to evaluate the pCR rate. The secondary objectives included 2‐year PFS, 2‐year OS, the metabolic response to IC with trifluridine/tipiracil and oxaliplatin prior to standard CRT and following CRT prior to surgical resection as well as toxicities. Our exploratory objective was to correlate the ctDNA levels with disease recurrence and metabolic response on PET‐CT.

### 2.3 Eligibility

We enrolled patients with histologically proven EGAC who were between 18 and 76 years of age. Patients had potentially resectable endoscopic ultrasound–determined node‐positive disease with any T‐stage or T3–T4a with any N stage. Patients had an ECOG performance status (PS) of 0 or 1, and adequate bone marrow, liver, and kidney function. Patients had to be capable of taking oral tablet(s) without difficulty. Patients were excluded if they had known metastatic disease, T1N0, T2N0, or T4b disease, or if they received prior chemotherapy, radiotherapy, or prior surgical resection for EGAC. Patients with Grade 2 or higher peripheral neuropathy, or Grade 3 or higher hypersensitivity reaction to oxaliplatin were also excluded. Patients who had prior treatment with trifluridine/tipiracil, major surgery, or field radiation within 4 weeks prior to entering the study were also not allowed to participate.

### 2.4 Study Treatment and Procedures

Patients meeting the inclusion criteria received three cycles of trifluridine/tipiracil (35 mg/m^2^ BID on Days 1–5) and oxaliplatin (85 mg/m^2^) every 2 weeks before undergoing concurrent CRT (radiation dose of 5040 cGY was utilized at all participating study sites) with weekly carboplatin (AUC 2) and paclitaxel (50 mg/m^2^) for 6 weeks, followed by surgery (Figure S1).

Patients had FDG‐PET‐CT at baseline and within 2 weeks of completion of IC. Blood samples for retrospective correlative studies were collected and banked using two, 10‐mL Streck Cell‐free DNA BCT tubes at baseline, after completion of IC (within 2 weeks), after completion of CRT (within 2 weeks), after surgical resection (within 3 days of procedure), and after disease recurrence (within 3 days of confirmed recurrence).

### 2.5 Study Endpoints

The primary endpoint of the study was pCR rate as measured by tumor regression score. Secondary endpoints included 2‐year PFS, 2‐year OS rate, and metabolic response to IC as measured by the change in maximum standardized uptake value (SUVmax) on PET‐CT scans from pre‐ to post‐IC. PFS was defined as the time from initiation of IC to either disease progression or death from any cause. OS was defined as the time from initiation of IC to death from any cause. Patients with a > 35% decrease in SUVmax were categorized as metabolic responders, and those with a ≤ 35% decrease in SUVmax were categorized as metabolic non‐responders.

### 2.6 Exploratory Analysis: ctDNA Clearance

ctDNA is the DNA released from tumor cells into the peripheral circulation. The objective of the exploratory analysis was to quantify ctDNA in peripheral blood samples and correlate it with survival metrics (OS, PFS), tumor TNM stage, tumor regression score, and PET response. ctDNA levels were assessed at prespecified time points including baseline, after IC, after CRT, and after surgery. ctDNA clearance was defined as negative ctDNA levels after surgery, and the impact of ctDNA clearance status on PFS and OS was assessed, respectively.

### 2.7 Personalized ctDNA Assay

A clinically validated, personalized, tumor‐informed 16‐plex polymerase chain reaction (mPCR)–next‐generation sequencing assay (Signatera, Natera Inc.) was used for the detection and quantification of ctDNA in blood samples as previously described [19]. Briefly, formalin‐fixed, paraffin‐embedded tumor tissue from surgical resection or biopsy samples and matched normal DNA extracted from peripheral blood samples were processed for whole‐exome sequencing to identify and track up to 16 tumor‐specific somatic single nucleotide variants (SNVs) in the associated patient's plasma. Plasma was isolated from blood collected in Streck cfDNA BCT. Cell‐free DNA was extracted using the Qiagen QIAsymphony Circulating DNA Kit (10 mL) from patient plasma (median 7.7 mL; range 1.5–10.2 mL) at a given time point and was used to detect ctDNA using the personalized tumor‐informed assay. Plasma samples with at least two tumor‐specific variants detected above a predefined threshold were defined as ctDNA positive. ctDNA concentration was reported as mean tumor molecules/mL of plasma.

### 2.8 Safety

Toxicity was evaluated by monitoring adverse events (AEs) in response to any treatment modality as per CTCAE v5.0 and were summarized by attribution and grade using frequencies and relative frequencies. All patients who received at least one dose of any of the study drugs (tipiracil/trifluridine, oxaliplatin, carboplatin, paclitaxel), radiation therapy, or surgery were evaluated for AEs. We reported any AEs that occurred at a frequency of more than 20% and all Grade 3 or higher AEs.

### 2.9 Statistical Considerations

The primary outcome of the study was pCR. The definition of pCR and near pCR was according to the modified Ryan scheme; pCR is defined as no viable cancer cells, and near pCR is defined as single cells or rare small groups of cancer cells in a surgical specimen [20, 21]. Historically, the pCR rate in this patient population is approximately 20%. We expected the pCR rate after IC to increase by 15% based on the study by Goodman et al. [9]. The sample size calculations were based on the primary analysis, which evaluated the one‐sided hypotheses about the actual pCR rate using the Simon two‐stage minimax design.

#### 2.9.1 Sample Size Calculation

The study had Simon's minimax design with a plan to enroll 22 patients (*n*1) in Stage 1. If four or fewer pCRs were observed, the study would terminate, and the treatment would not be considered promising; otherwise, an additional *n*2 = 19 patients would be enrolled in Stage 2. If 12 or more pCRs were observed, the treatment would be considered promising. If the actual pCR rate was = 0.35, then the study design (*n* = *n*1 + *n*2 = 22 + 19 = 41 evaluable patients) would achieve 80.4% power at a significance level of *α* = 0.1. To account for non‐evaluable patients, up to *n* = 45 patients were planned to be enrolled.

Patients without a response assessment who discontinued treatment due to reasons other than treatment toxicity or disease progression were considered non‐evaluable for the primary analysis and were replaced. The overall and disease‐specific survival were summarized using standard Kaplan–Meier methods, where estimates of median survival and 2‐year survival rates were obtained with 90% confidence intervals. Descriptive statistics (as appropriate: number, percent, mean, median, min, and max) were used to summarize demographic and baseline characteristics.

## 3 Results

### 3.1 Patient Characteristics

A total of 22 patients with any node‐positive or T3–T4a N0 EGAC were enrolled at our centers between December 20, 2019 and August 29, 2022 and started chemotherapy with 3 cycles of trifluridine/tipiracil and oxaliplatin and concurrent CRT with carboplatin and paclitaxel (Figure 1). The median age of enrolled patients was 61.3 years (range: 27.51–75.33) and 19 (86.4%) patients were male. Regarding the primary disease site, 54.5% of cases were GEJ, and 45.5% of cases were esophagus. Twelve patients (54.55%) had histology Grade 2, and ten patients (45.45%) had Grade 3 disease. Seven (31.8%) patients had no node disease, whereas 15 (68.18%) out of the total 22 patients were node‐positive (Table 1).

**TABLE 1:** Patient characteristics.

		Overall
	*N*	22 (100%)
Age	Mean/Std/*N*	58.73/14.01/22
	Median/Min/Max	61.33/27.51/75.33
Race	White	20 (90.9%)
	Asian	1 (4.5%)
	Unknown	1 (4.5%)
Gender	Male	19 (86.4%)
	Female	3 (13.6%)
Performance status score (ECOG)	0	13 (59.1%)
	1	9 (40.9%)
Disease site	Gastroesophageal junction	12 (54.5%)
	Esophagus	10 (45.5%)
Histology grade	Grade II	12 (54.55%)
	Grade III	10 (45.45%)
T‐stage	II	2 (9.1%)
	III	20 (90.9%)
N‐stage	0	7 (31.8%)
	I	4 (18.2%)
	II	8 (36.4%)
	III	3 (13.6%)

Among 22 total study participants, two had disease progression and two had AEs prior to CRT, including bowel perforation from known diverticulitis for which the IC was not attributable, and prolonged neutropenia was observed after two cycles of IC. Thus, 18 patients completed CRT, following which two patients had disease progression and one patient had an AE. Among the 18 patients who completed CRT, 11 patients completed all six cycles of carboplatin plus paclitaxel at the protocol‐defined dose, and none required dose reduction. The remaining seven patients did not complete six cycles; four received five cycles, two received four cycles, and one patient had two episodes of infusion‐related reactions followed by persistent anemia after one cycle of treatment and was taken off the treatment. Surgery in one patient was terminated due to unresectable disease determined during the procedure. In total, 14 patients underwent surgery with R0 resection and completed the study (Figure 1).

### 3.2 Treatment Response and Survival

The primary endpoint was the pCR rate to therapy measured by tumor regression score. Seven (50.0%) had partial, five (35.7%) had near complete, and two (14.3%) had complete tumor regression. Age, ECOG PS, and nodal stage were not associated with pathological response outcomes (Table S1). Mean of baseline SUVmax for 22 patients was 13.99. Mean SUVmax at induction was decreased by 32.69% in 20 evaluable patients. Twelve patients (60%) were metabolic responders after IC. Mean SUVmax after completion of CRT was decreased by 56.47% from baseline in seven evaluable patients (Table S2). PET‐CT SUVmax at various time points is illustrated in Figure S2.

**FIGURE 2:**
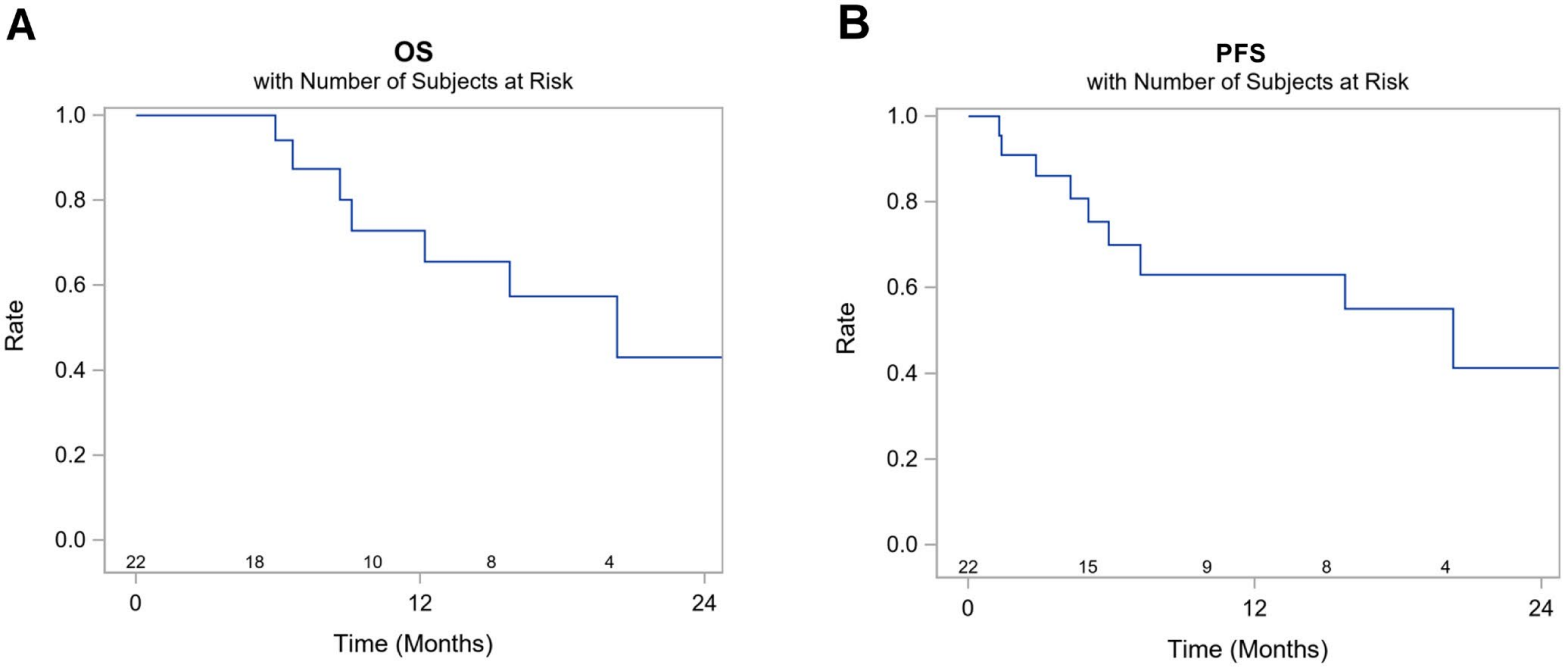
Kaplan–Meier plots of overall survival (A) and progression‐free survival (B) (*N* = 22). OS, overall survival; PFS, progression‐free survival.

The 12‐month and 24‐month OS rates were 73% (95% confidence interval [CI] 42–89) and 43% (95% CI 14–70), respectively, and the median OS was 20.3 months (95% CI 8.6‐Not reached [NR]). The 12‐ and 24‐month PFS rates were 63% (95% CI 37–81) and 41% (95% CI 0.14–0.68), respectively, and the median PFS was 20.3 months (95% CI 5.9‐NR) (Figure 2).

### 3.3 Exploratory Analysis: ctDNA Analysis

**FIGURE 3:**
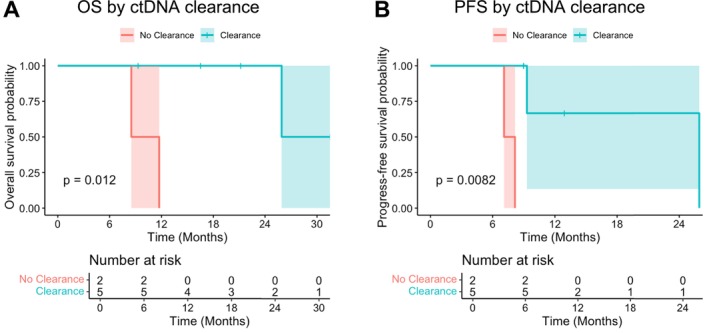
Kaplan–Meier plots of overall survival (A) and progression‐free survival (B) by ctDNA clearance (*N* = 7). Green label indicates patients with ctDNA clearance and red label indicates those without ctDNA clearance. ctDNA clearance was associated with (A) longer OS (median OS 25.9 vs. 10.1 months, *p* = 0.012) and (B) longer PFS (median PFS 25.9 vs. 7.6 months, *p* = 0.008). ctDNA, circulating tumor DNA; OS, overall survival; PFS, progression‐free survival.

**FIGURE 4:**
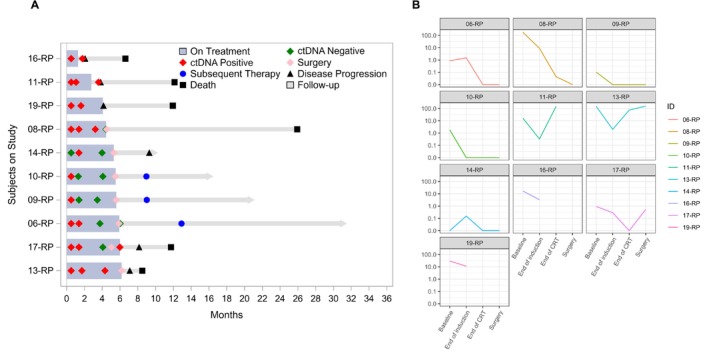
Swimmer's plot of patient outcomes and changes in ctDNA expression. (A) Swimmer's plot illustrates clinical outcomes of 10 patients with ctDNA information available for analysis. (B) Changes in ctDNA expression throughout the treatment course in each patient. *X*‐axis: evaluation point; *Y*‐axis: ctDNA expression level (MTM/mL). CRT, chemoradiotherapy; ctDNA, circulating tumor DNA.

Out of 22 patients, 10 patients had available information on ctDNA kinetics throughout the treatment course (Table S3). ctDNA expression kinetics at baseline, after IC, after CRT, and after surgery based on clinical characteristics are available in Tables S4, S5 and Figure S3, and their correlations with SUV are summarized in Figure S4. Among these 10 patients, seven patients were eligible for OS and PFS analysis based on ctDNA clearance status at the time of surgery. Of the seven patients eligible for OS and PFS analyses, patients who cleared ctDNA (ctDNA‐negative post‐surgery, *N* = 5) had significantly higher OS (*p* = 0.012) and PFS (*p* = 0.0082) than those without ctDNA clearance (*N* = 2) (Figure 3). Post‐op ctDNA‐positive results predicted poorer OS compared to those with ctDNA‐negative results (*p* = 0.0096). Pre‐op and post‐CRT ctDNA‐positive results predicted poorer PFS compared to those with ctDNA‐negative results (*p* = 0.0046) (Table S6). Negative nodal disease prior to IC was associated with achieving ctDNA clearance, but other clinical factors including tumor response score, T stage, and SUV response were not strongly correlated with ctDNA clearance status (Figure S5). OS and PFS in all 10 patients and according to radiological or pathological response after IC and CRT are illustrated in Figure S6. Details of clinical outcomes including ctDNA status, disease progression, and treatment sequence in each patient are available in Figure 4.

### 3.4 Safety

Among all treated patients (*n* = 22), nausea and fatigue were the most common AEs of any grade, both occurring in 13 (59.1%) subjects. Constipation (50%), diarrhea (40.9%), and vomiting (36.4%) were also common AEs (Table 2). Neutropenia was the most common Grade 3 or more AEs seen, occurring in two (9.1%) patients, while mediastinal infection and syncope occurred in one (4.5%) patient, respectively. Safety information of this study is summarized in Table S7.

**TABLE 2:** Adverse events attributable to treatment with a frequency of 20% or more.

Adverse event summary (*n* = 22)		Any AEs *n* (%)
System organ class	Preferred term	
Gastrointestinal disorders	Constipation	11 (50%)
	Diarrhea	9 (40.9%)
	Nausea	13 (59.1%)
	Vomiting	8 (36.4%)
General disorders and administration site conditions	Fatigue	13 (59.1%)
Investigations	Neutropenia	5 (22.7%)
Metabolism and nutrition disorders	Anorexia	6 (27.3%)
Nervous system disorders	Dysesthesia	5 (22.7%)
	Dysgeusia	5 (22.7%)
	Peripheral sensory neuropathy	7 (31.8%)

Abbreviation: AEs, adverse events.

### 3.5 Follow‐Up Data on Disease Recurrence

On follow‐up as of August 2024, seven (50.0%) of the 14 patients who underwent surgery had recurrence. Median days of recurrence after surgery were 120 days (range 28–730 days). Liver (*N* = 3, 42.9%) and brain (*N* = 2, 28.6%) were the most common sites of recurrence. Other recurrent sites were the lung, femoral neck, thoracic lymph nodes, and peritoneum (Table S8).

## 4 Discussion

This single‐arm, Phase II study evaluating the efficacy of IC with three cycles of trifluridine/tipiracil and oxaliplatin followed by CRT and surgery in high‐risk EGAC did not meet its primary endpoint of improving pCR rate compared to historical reports. Interestingly, the feasibility of the induction therapy was shown, and there was a correlation between ctDNA clearance and improved survival (median OS 25.9 vs. 10.1 months, *p* = 0.012), which merits further investigation in patients with locally advanced EGAC who are candidates for multimodality therapy.

For more than a decade, CRT with carboplatin and paclitaxel followed by surgery has been a standard of care in resectable esophageal/GEJ cancer after the CROSS trial showed an improvement in OS by adding neoadjuvant CRT prior to surgery [6]. However, a recent long‐term follow‐up of the CROSS trial showed that the rate of distant metastasis was not different between the CRT arm vs. the surgery alone arm, and the benefit of CRT appeared mainly related to reducing locoregional relapse likely due to the fact that the chemotherapy administered during CRT is low‐dose and, therefore, is not very effective in controlling distant metastasis [6]. For that reason, some studies have used IC prior to starting CRT to treat micrometastatic disease as well as to downstage the tumor. Our study showed the feasibility and safety of the strategy employing IC followed by CRT prior to surgery. Compared to our intuitional data and previous trials assessing preoperative CRT, IC did not compromise the R0 resection rate or increase the surgical complication rate (Table S9) [5, 22, 23, 24]. Notably, we observed PET response with IC in 60% of patients, although the primary endpoint of improved pCR with IC was not met. Our findings are comparable to another study where IC did not improve the pathological response rate [8]; however, the CALGB80803 trial showed improved pCR with IC [9]. The unique aspect of that study was the use of a risk‐adaptive strategy that involved changing the chemotherapy regimen during CRT based on PET response to IC. We did not modify our chemotherapy regimen, and all patients had the same chemotherapy regimen during CRT, which could explain the lack of pCR improvement in our study.

pCR has long been considered a surrogate marker for long‐term prognosis in some tumor types, such as breast cancer. One meta‐analysis before the era of immunotherapy revealed significantly improved PFS and OS in patients with gastric or GEJ adenocarcinoma who achieved pCR from neoadjuvant chemotherapy compared to those who did not have a pCR [25]. In contrast, pCR lacked a strong correlation with OS in an integrated study from 22 clinical trials, suggesting that pCR does not appear to serve as an adequate surrogate to replace PFS or OS [26]. This could be due to difficulty in preventing distant metastasis from CRT or neoadjuvant chemotherapy, and thus, clinical trials have recently incorporated immune checkpoint inhibitors into perioperative therapy.

In the KEYNOTE‐585 trial, the addition of pembrolizumab to cisplatin‐based chemotherapy or 5‐FU, leucovorin, oxaliplatin, and docetaxel (FLOT) was evaluated in locally advanced gastric and GEJ cancer. Although adding pembrolizumab improved pCR by 11%, it did not improve event‐free survival, suggesting that improved pCR may not predict survival in the neoadjuvant settings [27]. Other trials, such as the DANTE trial evaluating the addition of atezolizumab to perioperative FLOT and the MATTERHORN trial evaluating the addition of durvalumab to perioperative FLOT, demonstrated improvement in pCR rate by adding immunotherapy (by 9% in DANTE and by 12% in MATTERHORN trial). We await survival results to see if pCR status predicts survival in these studies [28, 29, 30].

One potential reason why there was no survival improvement in our study population is the failure to control micrometastasis leading to a high distant metastasis rate (50.0%). This is supported by the fact that we saw inferior PFS and OS in patients without ctDNA clearance. Our findings are consistent with the observations from the PLAGAST study, which evaluated longitudinal ctDNA levels during treatment of locally advanced resectable gastric or GEJ adenocarcinoma, revealing a strong association between ctDNA clearance and longer RFS and OS [31]. Patients with early ctDNA clearance had the best clinical outcomes in the PLAGAST study, suggesting that better control of microscopic metastasis would lead to prolonged survival. In our study, only pretreatment node status was significantly correlated with ctDNA clearance, suggesting lymph node status is an important clinical prognostic factor in locally advanced, resectable EGAC. This is supported by an analysis of the MAGIC trial evaluating the role of pCR and lymph node status after neoadjuvant chemotherapy in resectable gastric or GEJ adenocarcinoma. The investigators found that lymph node metastases, and not pCR, was the only independent predictor of survival after chemotherapy and surgery [32]. These results highlight that more unique and intensified strategies controlling microscopic metastasis from draining lymph are necessary in node‐positive populations with EGAC. Recent studies have investigated the utility of induction chemo‐immunotherapy followed by CRT with promising results in patients with locally advanced squamous cell esophageal cancer [33, 34]. More studies are needed in the adenocarcinoma population, focusing on the tumor immune microenvironment and providing a tailored approach incorporating immunotherapy to control microscopic metastasis [35].

There has been a long debate regarding the superiority between perioperative chemotherapy versus CRT and treatment sequence in resectable esophageal cancer. Both treatment strategies were comparable till the recent ESOPEC study, which randomized EGAC patients into perioperative chemotherapy following the FLOT protocol vs. neoadjuvant CRT from the CROSS protocol and reported improved pCR (19.3 vs. 13.5%) and OS (66 vs. 37 months, HR 0.70, 95% CI 0.53–0.92, *p* = 0.012) from perioperative FLOT [23]. Long‐term follow‐up data are awaited to compare locoregional and distant relapse‐free survival between perioperative FLOT and CROSS protocols. Additionally, the recently published TOPGEAR study demonstrated that the addition of CRT to preoperative chemotherapy compared to perioperative chemotherapy alone in patients with GEJ or gastric adenocarcinoma did not improve either PFS (31 vs. 32 months, HR 0.98, 95% CI 0.79–1.22) or OS (46 vs. 49 months, HR 1.05, 95% CI 0.83–1.31) despite a higher percentage of pCR in the preoperative chemotherapy plus CRT group (17% vs. 8%, difference 9% [95% CI 2–15]) [24]. Locoregional relapse or progression (37 vs. 33%) and distant relapse rate (44 vs. 42%) were both similar between chemotherapy plus CRT and chemotherapy alone groups [24]. These results raise the questions of whether pCR should be considered an endpoint in trials evaluating neoadjuvant therapy and the utility of CRT as a preoperative strategy for locally advanced upper gastrointestinal tract cancers. To further assess the utility of these neoadjuvant approaches, several studies evaluated these two strategies as a sequential therapy, including the TNT‐OES‐1 study demonstrating the feasibility and manageable toxicity profile from a FLOT‐CROSS sequence in oligometastatic EGAC. The ongoing TNT‐OES‐2 study will provide insight into the optimal sequencing of FLOT and CROSS protocols in the resectable population (NCT06161818). However, our results, taken together with those from ESOPEC and TOPGEAR studies, further suggest that preoperative CRT does not improve outcomes in resectable EGAC.

Our study has several limitations. First, we had a relatively small sample size of 22 patients, which limited survival analysis and subgroup analyses. Additionally, this was a single‐arm study, and thus, the impact of adding IC cannot be fully elucidated by comparing it to conventional CRT alone. Additionally, most participants in this study were Caucasian males, and thus, results should be interpreted with caution when applied to other demographics. Finally, although ctDNA‐positive results post‐surgery predicted reduced OS, longitudinal ctDNA samples were available for only a small group of patients. Nonetheless, our ctDNA results are consistent with numerous previous reports demonstrating the prognostic value of ctDNA status after definitive therapy for gastrointestinal and other malignancies. Future optimization and escalation studies that utilize ctDNA results will inform improved adjuvant treatment decision‐making and strategies to improve outcomes for patients with EGAC.

## 5 Conclusion

This Phase II study did not meet its primary endpoint of pCR improvement with an IC utilizing trifluridine/tipiracil and oxaliplatin prior to CRT in resectable EGAC. Given the findings from the ESOPEC and TOPGEAR studies, the perioperative FLOT regimen should remain the new standard of care in patients with resectable EGAC, and thus, CRT should have limited, if any, role in patients with resectable EGAC. Results of our study provide insight into the importance of controlling microscopic metastasis, particularly in those with nodal disease, as shown by the significant association between ctDNA clearance and survival outcomes. Future translational research to define biomarkers, along with improved radiation techniques, may help identify patient subgroups where radiation may be beneficial.

## Author Contributions

**Sarbajit Mukherjee:** conceptualization, methodology, validation, investigation, resources, writing – original draft, supervision, funding acquisition. **Yu Fujiwara:** investigation, project administration, writing – original draft, visualization. **Christos Fountzilas:** investigation, resources, writing – review and editing. **Harsha Pattnaik:** visualization, writing – original draft, investigation. **Sarah Chatley:** investigation, resources, writing – review and editing. **Deepak Vadehra:** investigation, writing – review and editing, resources. **Moshim Kukar:** investigation, resources, writing – review and editing. **Kristopher Attwood:** methodology, data curation, software, formal analysis, writing – review and editing. **Anthony George:** methodology, software, data curation, formal analysis, writing – review and editing. **Shailesh Advani:** investigation, resources, writing – review and editing. **Han Yu:** methodology, software, data curation, formal analysis, writing – review and editing. **Kayla Catalfamo:** investigation, writing – review and editing, resources. **Alyson Brown:** investigation, resources, writing – review and editing. **Erik Spickard:** methodology, software, data curation, formal analysis, writing – review and editing. **Arkarachai Fungtammasan:** software, methodology, data curation, formal analysis, writing – review and editing. **Sagila George:** investigation, resources, writing – review and editing. **Chih‐Yi Liao:** investigation, resources, writing – review and editing. **Renuka Iyer:** investigation, resources, writing – review and editing. **Hassan Hatoum:** investigation, writing – review and editing, resources.

## Ethics Statement

The study protocol was reviewed and approved by the Roswell Park Comprehensive Cancer Center institutional review board. The study was performed in accordance with the Declaration of Helsinki. All study participants consented to participate in the study by providing a written informed consent.

## Conflicts of Interest

Sarbajit Mukherjeeserves as a volunteer guidelines panel member at the National Comprehensive Cancer Network and American Society of Clinical Oncology. He received research funding from the National Comprehensive Cancer Network and Ipsen Biopharmaceuticals/North American Neuroendocrine Tumor Society, which were paid to the institute. S.M. received consulting fees from Merck, Eisai, and BeiGene Ltd. Yu Fujiwara: no conflicts of interest to declare. Y.F. receives expenses from Conquer Cancer, the ASCO Foundation, unrelated to this work. Christos Fountzilas: no conflicts of interest to declare. C.F. has funding from the National Cancer Institute, NCCN Oncology Research Program, NCCN Foundation, Pfizer Inc., Taiho Oncology, and Merck (all paid to institute and unrelated with this work). Harsha Pattnaik: no conflicts of interest to declare. Sarah Chatley: no conflicts of interest to declare. Deepak Vadehra: no conflicts of interest to declare. Moshim Kukar: no conflicts of interest to declare. Kristopher Attwood: no conflicts of interest to declare. Anthony George: no conflicts of interest to declare. Shailesh Advani: no conflicts of interest to declare. Han Yu: no conflicts of interest to declare. Kayla Catalfamo: no conflicts of interest to declare. Alyson Brown: no conflicts of interest to declare. Erik Spickard: Employee of Natera Inc. Arkarachai Fungtammasan: Employee of Natera Inc. Sagila George: receives research funding from Natera. Chih‐Yi Liaoserves as a consultant for Incyte, Exelixis, Lexicon, Ipsen, TransThera Biosciences, Blueprint Medicines, Genentech, QED Therapeutics, Histosonics, AstraZeneca, and Lilly. He participates in speakers' bureaus for Eisai, Exelixis, Incyte, and AstraZeneca, receives research funding from Bristol‐Myers Squibb, and receives travel/accommodations/expenses support from Exelixis, Eisai, Ipsen, Incyte, and AstraZeneca. Renuka Iyerserves as a consultant for Exelixis, Crinetics, Ipsen and receives grant support from Ipsen and TerSera. Hassan Hatoum: Immediate family member receives research funding from Natera. No other conflicts of interest to declare.

## Supporting information


Data S1.

## Data Availability

The data generated in this study are available upon request from the corresponding author.

## References

[1] K. E. L. McColl , “What Is Causing the Rising Incidence of Esophageal Adenocarcinoma in the West and Will It Also Happen in the East?,” Journal of Gastroenterology 54, no. 8 (2019): 669–673. 31172291 10.1007/s00535-019-01593-7 PMC6647360

[2] American Cancer Society , “Key Statistics for Esophageal Cancer,” (2024), https://www.cancer.org/cancer/esophageal‐cancer/about/key‐statistics.html.

[3] S. R. Demeester , “Epidemiology and Biology of Esophageal Cancer,” Gastrointest Cancer Res 3, no. 2 Suppl (2009): S2–S5. PMC2684731 19461918

[4] M. F. Berry , “Esophageal Cancer: Staging System and Guidelines for Staging and Treatment,” Journal of Thoracic Disease 6, no. Suppl 3 (2014): S289–S297. 24876933 10.3978/j.issn.2072-1439.2014.03.11 PMC4037413

[5] P. van Hagen , M. C. Hulshof , J. J. van Lanschot , et al., “Preoperative Chemoradiotherapy for Esophageal or Junctional Cancer,” New England Journal of Medicine 366, no. 22 (2012): 2074–2084. 22646630 10.1056/NEJMoa1112088

[6] B. M. Eyck , J. J. B. van Lanschot , M. Hulshof , et al., “Ten‐Year Outcome of Neoadjuvant Chemoradiotherapy Plus Surgery for Esophageal Cancer: The Randomized Controlled CROSS Trial,” Journal of Clinical Oncology 39, no. 18 (2021): 1995–2004, 10.1200/JCO.20.03614. 33891478

[7] J. V. Reynolds , S. R. Preston , B. O'Neill , et al., “Trimodality Therapy Versus Perioperative Chemotherapy in the Management of Locally Advanced Adenocarcinoma of the Oesophagus and Oesophagogastric Junction (Neo‐AEGIS): An Open‐Label, Randomised, Phase 3 Trial,” Lancet Gastroenterology & Hepatology 8, no. 11 (2023): 1015–1027. 37734399 10.1016/S2468-1253(23)00243-1 PMC10567579

[8] J. A. Ajani , L. Xiao , J. A. Roth , et al., “A Phase II Randomized Trial of Induction Chemotherapy Versus No Induction Chemotherapy Followed by Preoperative Chemoradiation in Patients With Esophageal Cancer,” Annals of Oncology 24, no. 11 (2013): 2844–2849. 23975663 10.1093/annonc/mdt339 PMC3937600

[9] K. A. Goodman , F. S. Ou , N. C. Hall , et al., “Randomized Phase II Study of PET Response‐Adapted Combined Modality Therapy for Esophageal Cancer: Mature Results of the CALGB 80803 (Alliance) Trial,” Journal of Clinical Oncology 39, no. 25 (2021): 2803–2815. 34077237 10.1200/JCO.20.03611 PMC8407649

[10] B. A. Weinberg , J. L. Marshall , and M. E. Salem , “Trifluridine/Tipiracil and Regorafenib: New Weapons in the War Against Metastatic Colorectal Cancer,” Clinical Advances in Hematology & Oncology 14, no. 8 (2016): 630–638. 27487107

[11] H. J. Lenz , S. Stintzing , and F. Loupakis , “TAS‐102, a Novel Antitumor Agent: A Review of the Mechanism of Action,” Cancer Treatment Reviews 41, no. 9 (2015): 777–783. 26428513 10.1016/j.ctrv.2015.06.001 PMC4624296

[12] K. Shitara , T. Doi , M. Dvorkin , et al., “Trifluridine/Tipiracil Versus Placebo in Patients With Heavily Pretreated Metastatic Gastric Cancer (TAGS): A Randomised, Double‐Blind, Placebo‐Controlled, Phase 3 Trial,” Lancet Oncology 19, no. 11 (2018): 1437–1448, 10.1016/S1470-2045(18)30739-3. 30355453

[13] Y. L. Verschoor , J. de Haarvan , J. G. den Bergvan , et al., “Neoadjuvant Atezolizumab Plus Chemotherapy in Gastric and Gastroesophageal Junction Adenocarcinoma: The Phase 2 PANDA Trial,” Nature Medicine 30, no. 2 (2024): 519–530. 10.1038/s41591-023-02758-x PMC10878980 38191613

[14] G. P. Botta , M. Abdelrahim , R. L. Drengler , et al., “Association of Personalized and Tumor‐Informed ctDNA With Patient Survival Outcomes in Pancreatic Adenocarcinoma,” Oncologist 29, no. 10 (2024): 859–869. 39022993 10.1093/oncolo/oyae155 PMC11449101

[15] B. M. Huffman , V. N. Aushev , G. L. Budde , et al., “Analysis of Circulating Tumor DNA to Predict Risk of Recurrence in Patients With Esophageal and Gastric Cancers,” JCO Precision Oncology 6 (2022): e2200420, 10.1200/PO.22.00420. 36480779 PMC10530958

[16] E. Fontana and E. Ignatova , “Mind the Target: Circulating Tumour DNA in Gastrointestinal Malignancies,” Current Opinion in Oncology 34, no. 4 (2022): 395–402. 35837709 10.1097/CCO.0000000000000846

[17] A. Pretta , E. Lai , C. Donisi , et al., “Circulating Tumour DNA in Gastrointestinal Cancer in Clinical Practice: Just a Dream or Maybe Not?,” World Journal of Clinical Oncology 13, no. 12 (2022): 980–983. 36618080 10.5306/wjco.v13.i12.980 PMC9813836

[18] O. B. Alese , N. Cook , A. Ortega‐Franco , M. B. Ulanja , L. Tan , and J. Tie , “Circulating Tumor DNA: An Emerging Tool in Gastrointestinal Cancers,” American Society of Clinical Oncology Educational Book 42 (2022): 1–20. 10.1200/EDBK_349143 35471832

[19] T. Reinert , T. V. Henriksen , E. Christensen , et al., “Analysis of Plasma Cell‐Free DNA by Ultradeep Sequencing in Patients With Stages I to III Colorectal Cancer,” JAMA Oncology 5, no. 8 (2019): 1124–1131. 31070691 10.1001/jamaoncol.2019.0528 PMC6512280

[20] R. Ryan , D. Gibbons , J. M. Hyland , et al., “Pathological Response Following Long‐Course Neoadjuvant Chemoradiotherapy for Locally Advanced Rectal Cancer,” Histopathology 47, no. 2 (2005): 141–146. 16045774 10.1111/j.1365-2559.2005.02176.x

[21] L. J. C. Burgart , V. William , D. Jain , and With guidance from the CAP Cancer and CAP Pathology Electronic Reporting Committees , Protocol for the Examination of Specimens From Patients With Carcinoma of the Esophagus. Version 4.2.0.1 (College of American Pathologists, 2022).

[22] Z. E. Stiles , B. L. Hagerty , M. Brady , S. Mukherjee , S. N. Hochwald , and M. Kukar , “Contemporary Outcomes for Resected Type 1‐3 Gastroesophageal Junction Adenocarcinoma: A Single‐Center Experience,” Journal of Gastrointestinal Surgery 28, no. 5 (2024): 634–639. 38704200 10.1016/j.gassur.2024.01.040

[23] J. Hoeppner , T. Brunner , F. Lordick , et al., “Prospective Randomized Multicenter Phase III Trial Comparing Perioperative Chemotherapy (FLOT Protocol) to Neoadjuvant Chemoradiation (CROSS Protocol) in Patients With Adenocarcinoma of the Esophagus (ESOPEC Trial),” Journal of Clinical Oncology 42, no. 17_suppl (2024): LBA1. 10.1186/s12885-016-2564-y PMC4952147 27435280

[24] T. Leong , B. M. Smithers , M. Michael , et al., “Preoperative Chemoradiotherapy for Resectable Gastric Cancer,” New England Journal of Medicine 391 (2024): 1810–1821. 39282905 10.1056/NEJMoa2405195

[25] Z. Li , F. Shan , Y. Wang , et al., “Correlation of Pathological Complete Response With Survival After Neoadjuvant Chemotherapy in Gastric or Gastroesophageal Junction Cancer Treated With Radical Surgery: A Meta‐Analysis,” PLoS One 13, no. 1 (2018): e0189294. 29370182 10.1371/journal.pone.0189294 PMC5784899

[26] F. Petrelli , G. Tomasello , and S. Barni , “Surrogate End‐Points for Overall Survival in 22 Neoadjuvant Trials of Gastro‐Oesophageal Cancers,” European Journal of Cancer 76 (2017): 8–16. 28262586 10.1016/j.ejca.2017.01.032

[27] K. Shitara , S. Y. Rha , L. S. Wyrwicz , et al., “Neoadjuvant and Adjuvant Pembrolizumab Plus Chemotherapy in Locally Advanced Gastric or Gastro‐Oesophageal Cancer (KEYNOTE‐585): An Interim Analysis of the Multicentre, Double‐Blind, Randomised Phase 3 Study,” Lancet Oncology 25, no. 2 (2024): 212–224. 38134948 10.1016/S1470-2045(23)00541-7

[28] S. Lorenzen , T. O. Götze , P. Thuss‐Patience , et al., “Perioperative Atezolizumab Plus Fluorouracil, Leucovorin, Oxaliplatin, and Docetaxel for Resectable Esophagogastric Cancer: Interim Results From the Randomized, Multicenter, Phase II/III DANTE/IKF‐s633 Trial,” Journal of Clinical Oncology 42, no. 4 (2024): 410–420, 10.1200/JCO.23.00975. 37963317

[29] D. Y. Oh , Y. Y. Janjigian , S. E. Al‐Batran , et al., “129O Pathological Complete Response (pCR) to Durvalumab Plus 5‐Fluorouracil, Leucovorin, Oxaliplatin and Docetaxel (FLOT) in Resectable Gastric and Gastroesophageal Junction Cancer (GC/GEJC): Interim Results of the Global, Phase III MATTERHORN Study,” Annals of Oncology 34 (2023): S1520.

[30] Y. Y. Janjigian , S.‐E. Al‐Batran , Z. A. Wainberg , et al., “Pathological Complete Response (pCR) to 5‐Fluorouracil, Leucovorin, Oxaliplatin and Docetaxel (FLOT) With or Without Durvalumab (D) in Resectable Gastric and Gastroesophageal Junction Cancer (GC/GEJC): Subgroup Analysis by Region From the Phase 3, Randomized, Double‐Blind MATTERHORN Study,” Journal of Clinical Oncology 42, no. 3_suppl (2024): LBA246.

[31] A. Zaanan , A. Didelot , C. Broudin , et al., “Longitudinal Circulating Tumor DNA (ctDNA) Analysis During Treatment (Tx) of Locally Advanced Resectable (LAR) Gastric or Gastroesophageal Junction (G/GEJ) Adenocarcinoma (ADENOCA): The PLAGAST Prospective Biomarker Study,” Journal of Clinical Oncology 42, no. 16_suppl (2024): 4028, 10.1200/JCO.2024.42.16_suppl.4028. PMC12289871 40707450

[32] E. C. Smyth , M. Fassan , D. Cunningham , et al., “Effect of Pathologic Tumor Response and Nodal Status on Survival in the Medical Research Council Adjuvant Gastric Infusional Chemotherapy Trial,” Journal of Clinical Oncology 34, no. 23 (2016): 2721–2727. 27298411 10.1200/JCO.2015.65.7692 PMC5019747

[33] D. Ai , S. Hao , W. Shen , et al., “Induction Sintilimab and Chemotherapy Followed by Concurrent Chemoradiotherapy for Locally Advanced Esophageal Cancer: A Proof‐Of‐Concept, Single‐Arm, Multicenter, Phase 2 Trial,” EClinicalMedicine 69 (2024): 102471. 38356729 10.1016/j.eclinm.2024.102471 PMC10864194

[34] H. M. Lian , J. L. Wu , W. J. Liufu , et al., “Induction Immunotherapy Plus Chemotherapy Followed by Definitive Chemoradiation Therapy in Locally Advanced Esophageal Squamous Cell Carcinoma: A Propensity‐Score Matched Study,” Cancer Immunology, Immunotherapy 73, no. 3 (2024): 55. 38366287 10.1007/s00262-024-03649-x PMC10873219

[35] Y. Vedire , S. Kalvapudi , R. J. Seager , et al., “Immune Checkpoint Expression and Co‐Expression Landscape in Gastroesophageal Adenocarcinoma,” ESMO Gastrointestinal Oncology 3 (2024): 100045. 10.1016/j.esmogo.2024.100045 PMC12836530 41648743

