# Supplementary material for: Trifluridine/Tipiracil and Oxaliplatin as Induction Chemotherapy in Resectable Esophageal and Gastroesophageal Junction Adenocarcinoma: A Phase II Study
Source: Cancer Med. 2025 Apr 8;14(7):e70835. doi: 10.1002/cam4.70835 (PMC11978735; doi:10.1002/cam4.70835)
Supplement: Supplementary file 1 — Data S1. [file CAM4-14-e70835-s001.docx]

**Supplementary material**

**Trifluridine/Tipiracil and Oxaliplatin as Induction Chemotherapy in Resectable Esophageal and Gastroesophageal Junction Adenocarcinoma: A Phase II Study**

Authors: Sarbajit Mukherjee, Yu Fujiwara, Christos Fountzilas, Harsha Pattnaik, Sarah Chatley, Deepak Vadehra, Moshim Kukar, Kristopher Attwood, Anthony George, Shailesh Advani, Han Yu, Kayla Catalfamo, Alyson Brown, Erik Spickard, Arkarachai Fungtammasan, Sagila George, Chih-Yi Liao, Renuka Iyer, Hassan Hatoum

Index

Table S1. Pathological response outcomes based on clinical factors

Table S2. Treatment response - PET response

Table S3. Clinical characteristics of 10 patients with ctDNA information

Table S4. ctDNA expression levels at prespecified timepoints

Table S5. Kinetics of ctDNA expression according to clinical factors: pathological response, T stage, N stage, and PET-CT response

Table S6: Univariate analysis of overall survival and progression-free survival according to ctDNA status at prespecified timepoints

Table S7. Adverse event summary

Table S8. Summary of surgical outcomes and complications in this study and other chemoradiation trials with an institutional reference

Table S9: Clinical data of patients with recurrence after surgery

Figure S1. Study schema

Figure S2: Summary of PET-CT findings at prespecified treatment timepoints

Figure S3: Changes in ctDNA expression over the treatment course

Figure S4: Correlations between SUV and ctDNA expression at prespecified different timepoints

Figure S5: Kaplan-Meier curves of overall survival and progression-free survival based on radiological and pathological response in 10 patients with ctDNA information

Figure S6: Kaplan-Meier curves of ctDNA clearance based on clinical factors

**Table S1. Pathological response outcomes based on clinical factors (N=14)**

| **Characteristic** | **Non pCR, N = 12 *^1^** | **pCR, N = 2 *^1^** | **p-value *^2^** |
| --- | --- | --- | --- |
| **Age** |  |  | 1.000 |
| **< 65** | 7 (58%) | 1 (50%) |  |
| **≥ 65** | 5 (42%) | 1 (50%) |  |
| **ECOG** |  |  | 1.000 |
| **0** | 8 (67%) | 1 (50%) |  |
| **1** | 4 (33%) | 1 (50%) |  |
| **N stage *^3^** |  |  | 0.5055 |
| **0** | 7 (58%) | 2 (100%) |  |
| **1-3** | 5 (42%) | 0 (0%) |  |
| **N stage *^4^** |  |  | 1.000 |
| **0-1** | 10 (83%) | 2 (100%) |  |
| **2-3** | 2 (17%) | 0 (0%) |  |

**Abbreviation:** ECOG, Eastern Cooperative Oncology Group; pCR, pathological completed response.

pCR listed here is equivalent to complete tumor regression described in the main text.

Non pCR means either partial or near complete tumor regression described in the main text.

*1 Number of patients (%)

*2 Fisher’s exact test

*3 N stage group by dividing patients into N0 and N1-3 stages

*4 N stage group by dividing patients into N0-1 and N2-3 stages

**Table S2: Treatment response - PET response**

|  | | **Overall** |
| --- | --- | --- |
|  | N | 22 (100.0) |
| Baseline SUVmax | Mean/Std/N | 13.99/7.82/22 |
|  | Median | 12.25 |
| Induction SUVmax | Mean/Std/N | 7.06/3.52/20 |
|  | Median | 5.44 |
| Chemoradiation SUVmax | Mean/Std/N | 2.14/2.69/7 |
|  | Median | 0.00 |
| Change at Induction (Induction - Baseline) | Mean/Std/N | -7.31/8.48/20 |
|  | Median | -5.10 |
| Change at Chemoradiation (Chemoradiation - Induction) | Mean/Std/N | -6.32/6.59/7 |
|  | Median | -5.42 |
| Percent Change at Induction | Mean/Std/N | -32.69/55.27/20 |
|  | Median | -38.84 |
| Percent Change at Chemoradiation | Mean/Std/N | -56.47/61.51/7 |
|  | Median | -100.00 |

**Abbreviation:** SUV, standardized uptake value.

**Table S3. Clinical characteristics of 10 patients with ctDNA information**

|  |  | **N** |
| --- | --- | --- |
|  |  | **10** |
| **Age** | Mean (SD) | 64.17 (11.15) |
|  | Median (IQR) | 68.41 (60.62, 70.22) |
| **Race** | White | 9 (90.00%) |
|  | Unknown | 1 (10.00%) |
| **Sex** | Male | 8 (80.00%) |
|  | Female | 2 (20.00%) |
| **T-Stage** | 2 | 1 (10.00%) |
|  | 3 | 9 (90.00%) |
| **N-Stage** | 0 | 6 (60.00%) |
|  | 1+ | 4 (40.00%) |

**Abbreviations:** ctDNA, circulating tumor DNA; IQR, interquartile range; SD, standard deviation.

**Table S4. ctDNA expression levels at prespecified timepoints**

|  | **ctDNA Expression** | |
| --- | --- | --- |
|  | **Mean (SD)** | **Median (IQR)** |
| **Baseline** | 39.62 (67.12) | 9.029 (0.8839, 26.44) |
| **Induction** | 2.774 (4.04) | 0.9194 (0.1875, 2.955) |
| **CRT** | 27.25 (53.76) | 0.0 (0.0, 18.57) |
| **Surgery** | 22.23 (58.59) | 0.0 (0.0, 0.2717) |
| **Overall** | 22.79 (51.03) | 0.3294 (0.0, 10.1) |

**Abbreviations:** ctDNA, circulating tumor DNA; CRT, chemoradiotherapy; IQR, interquartile range; SD, standard deviation.

**Table S5. Kinetics of ctDNA expression according to clinical factors: tumor response score (pathological response), T stage, N stage, and PET-CT response**

**(A) Tumor response score**

| **Tumor Response Score** | | **Near Complete Response** | **Partial Response** | **Overall** | **P-Value** |
| --- | --- | --- | --- | --- | --- |
|  |  | **2** | **5** | **7** |  |
| **Baseline ctDNA** | Mean (SD) | 0.8991 (0.04287) | 66.36 (90.56) | 47.66 (80.55) | 0.8571 |
|  | Median (IQR) | 0.8991 (0.8839, 0.9142) | 1.808 (0.1031, 150.8) | 0.9294 (0.4859, 76.3) |  |
| **Induction ctDNA** | Mean (SD) | 0.9 (0.8618) | 2.212 (3.845) | 1.837 (3.224) | 0.8451 |
|  | Median (IQR) | 0.9 (0.5953, 1.205) | 0.1531 (0.0, 1.983) | 0.2906 (0.07656, 1.746) |  |
| **CRT ctDNA** | Mean (SD) | 0.0 (0.0) | 14.84 (33.15) | 10.6 (28.08) | 0.4687 |
|  | Median (IQR) | 0.0 (0.0, 0.0) | 0.0 (0.0, 0.0425) | 0.0 (0.0, 0.02125) |  |
| **Surgery ctDNA** | Mean (SD) | 0.2717 (0.3842) | 31.02 (69.36) | 22.23 (58.59) | 0.8091 |
|  | Median (IQR) | 0.2717 (0.1358, 0.4075) | 0.0 (0.0, 0.0) | 0.0 (0.0, 0.2717) |  |

**(B) T stage**

| **T-Stage** | | **2** | **3** | **Overall** | **P-Value** |
| --- | --- | --- | --- | --- | --- |
|  |  | **1** | **9** | **10** |  |
| **Baseline ctDNA** | Mean (SD) | 16.66 (NA) | 42.17 (70.68) | 39.62 (67.12) |  |
|  | Median (IQR) | 16.66 (16.66, 16.66) | 1.808 (0.8688, 29.7) | 9.029 (0.8839, 26.44) |  |
| **Induction ctDNA** | Mean (SD) | 3.279 (NA) | 2.718 (4.281) | 2.774 (4.04) |  |
|  | Median (IQR) | 3.279 (3.279, 3.279) | 0.3294 (0.1531, 1.983) | 0.9194 (0.1875, 2.955) |  |
| **CRT ctDNA** | Mean (SD) |  | 27.25 (53.76) | 27.25 (53.76) |  |
|  | Median (IQR) |  | 0.0 (0.0, 18.57) | 0.0 (0.0, 18.57) |  |
| **Surgery ctDNA** | Mean (SD) |  | 22.23 (58.59) | 22.23 (58.59) |  |
|  | Median (IQR) |  | 0.0 (0.0, 0.2717) | 0.0 (0.0, 0.2717) |  |

**(C) N stage**

| **N-Stage** | | **0** | **1+** | **Overall** | **P-Value** |
| --- | --- | --- | --- | --- | --- |
|  |  | **6** | **4** | **10** |  |
| **Baseline ctDNA** | Mean (SD) | 30.47 (72.82) | 53.35 (65.26) | 39.62 (67.12) | 0.1143 |
|  | Median (IQR) | 0.8991 (0.2945, 1.588) | 23.18 (16.56, 59.97) | 9.029 (0.8839, 26.44) |  |
| **Induction ctDNA** | Mean (SD) | 1.813 (3.531) | 4.215 (4.855) | 2.774 (4.04) | 0.1087 |
|  | Median (IQR) | 0.2219 (0.03828, 1.205) | 2.631 (1.57, 5.277) | 0.9194 (0.1875, 2.955) |  |
| **CRT ctDNA** | Mean (SD) | 0.007083 (0.01735) | 109.0 (49.24) | 27.25 (53.76) | **0.0357** |
|  | Median (IQR) | 0.0 (0.0, 0.0) | 109.0 (91.55, 126.4) | 0.0 (0.0, 18.57) |  |
| **Surgery ctDNA** | Mean (SD) | 0.09056 (0.2218) | 155.1 (NA) | 22.23 (58.59) | 0.119 |
|  | Median (IQR) | 0.0 (0.0, 0.0) | 155.1 (155.1, 155.1) | 0.0 (0.0, 0.2717) |  |

**(D) PET-CT response**

| **SUV Response** | | **No** | **Yes** | **Overall** | **P-Value** |
| --- | --- | --- | --- | --- | --- |
|  |  | **2** | **8** | **10** |  |
| **Baseline ctDNA** | Mean (SD) | 90.02 (126.0) | 27.02 (51.17) | 39.62 (67.12) | 0.5333 |
|  | Median (IQR) | 90.02 (45.48, 134.6) | 9.029 (0.6773, 19.92) | 9.029 (0.8839, 26.44) |  |
| **Induction ctDNA** | Mean (SD) | 4.608 (6.106) | 2.315 (3.802) | 2.774 (4.04) | 0.6944 |
|  | Median (IQR) | 4.608 (2.449, 6.767) | 0.9194 (0.1148, 2.307) | 0.9194 (0.1875, 2.955) |  |
| **CRT ctDNA** | Mean (SD) | 0.02125 (0.03005) | 36.32 (60.42) | 27.25 (53.76) | 1.00 |
|  | Median (IQR) | 0.02125 (0.01062, 0.03188) | 0.0 (0.0, 55.61) | 0.0 (0.0, 18.57) |  |
| **Surgery ctDNA** | Mean (SD) | 0.2717 (0.3842) | 31.02 (69.36) | 22.23 (58.59) | 0.8091 |
|  | Median (IQR) | 0.2717 (0.1358, 0.4075) | 0.0 (0.0, 0.0) | 0.0 (0.0, 0.2717) |  |

**Abbreviations:** IQR, interquartile range; N/A, not available; SD, standard deviation.

**Table S6: Univariate analysis of overall survival and progression-free survival according to ctDNA status at prespecified timepoints**

**(A) Overall survival**

| **Overall Survival** | **HR** | **2.50%** | **97.50%** | **P-Value** |
| --- | --- | --- | --- | --- |
| **ctDNA at Baseline** | 1.003 | 0.9931 | 1.014 | 0.5302 |
| **ctDNA at Induction** | 1.061 | 0.8844 | 1.272 | 0.5344 |
| **ctDNA at CRT** | 1.011 | 0.9949 | 1.028 | 0.1968 |
| **ctDNA at Surgery** | 188.4 | 0.01859 | 1.91E+06 | **0.0096792** |
| **Time to Clearance** | 1.576 | 0.7183 | 3.457 | 0.2262 |

**(B) Progression-free survival**

| **Progression-Free Survival** | **HR** | **2.50%** | **97.50%** | **P-Value** |
| --- | --- | --- | --- | --- |
| **ctDNA at Baseline** | 0.9982 | 0.9861 | 1.010 | 0.7644 |
| **ctDNA at Induction** | 1.019 | 0.8286 | 1.253 | 0.8609 |
| **ctDNA at CRT** | 1.147 | 0.2390 | 5.509 | **0.0045648** |
| **ctDNA at Surgery** |  |  |  | **< 2.2E-16** |
| **Time to Clearance** | 1.213 | 0.6433 | 2.286 | 0.5447 |

**Abbreviations:** ctDNA, circulating tumor DNA; CRT, chemoradiotherapy; HR, hazard ratio.

**Table S7. AE Summary**

**Table S7.1: Attribution to Study Treatment 1 (Carboplatin or Trifluridine & Tipiracil Hydrochloride)**

***Type and Degree for Non-Severe Adverse Event***

***(Total N Treated=22)***

| **Adverse Event** | | **Grade** | | | |
| --- | --- | --- | --- | --- | --- |
|  |  | 1 | 2 | 3 | 4 |
| **System Organ Class** | **Preferred Term** | 1 | 1 | 0 | 0 |
| Blood and lymphatic system disorders | **Any AE - Maximum Grade Seen |  |  |  |  |
|  | Anemia | 1 | 1 | 0 | 0 |
| Gastrointestinal disorders | **Any AE - Maximum Grade Seen | 8 | 7 | 1 | 0 |
|  | Abdominal pain | 1 | 1 | 1 | 0 |
|  | Bloating | 1 | 0 | 0 | 0 |
|  | Constipation | 9 | 1 | 0 | 0 |
|  | Diarrhea | 7 | 2 | 0 | 0 |
|  | Dry mouth | 1 | 0 | 0 | 0 |
|  | Dyspepsia | 0 | 2 | 0 | 0 |
|  | Gastritis | 0 | 1 | 0 | 0 |
|  | Mucositis oral | 2 | 0 | 0 | 0 |
|  | Nausea | 7 | 6 | 0 | 0 |
|  | Oral hemorrhage | 1 | 0 | 0 | 0 |
|  | Vomiting | 4 | 4 | 0 | 0 |
| General disorders and administration site conditions | **Any AE - Maximum Grade Seen | 7 | 6 | 0 | 0 |
|  | Fatigue | 8 | 5 | 0 | 0 |
|  | Fever | 0 | 1 | 0 | 0 |
|  | Flu like symptoms | 1 | 0 | 0 | 0 |
|  | Malaise | 1 | 0 | 0 | 0 |
| Injury, poisoning and procedural complications | **Any AE - Maximum Grade Seen | 0 | 2 | 0 | 0 |
|  | Infusion related reaction | 0 | 2 | 0 | 0 |
| Investigations | **Any AE - Maximum Grade Seen | 1 | 2 | 3 | 1 |
|  | Alanine aminotransferase increased | 1 | 0 | 0 | 0 |
|  | Aspartate aminotransferase increased | 1 | 0 | 0 | 0 |
|  | Lymphocyte count decreased | 0 | 0 | 2 | 0 |
|  | Neutrophil count decreased | 0 | 2 | 2 | 1 |
|  | Platelet count decreased | 1 | 2 | 0 | 0 |
|  | Weight loss | 1 | 0 | 0 | 0 |
|  | White blood cell decreased | 0 | 1 | 1 | 0 |
| Metabolism and nutrition disorders | **Any AE - Maximum Grade Seen | 4 | 2 | 0 | 0 |
|  | Anorexia | 5 | 1 | 0 | 0 |
|  | Dehydration | 0 | 1 | 0 | 0 |
| Musculoskeletal and connective tissue disorders | **Any AE - Maximum Grade Seen | 1 | 0 | 0 | 0 |
|  | Back pain | 1 | 0 | 0 | 0 |
| Nervous system disorders | **Any AE - Maximum Grade Seen | 7 | 1 | 1 | 0 |
|  | Dizziness | 1 | 0 | 0 | 0 |
|  | Dysesthesia | 2 | 0 | 0 | 0 |
|  | Dysgeusia | 5 | 0 | 0 | 0 |
|  | Headache | 0 | 1 | 0 | 0 |
|  | Peripheral sensory neuropathy | 1 | 0 | 0 | 0 |
|  | Syncope | 0 | 0 | 1 | 0 |
| Renal and urinary disorders | **Any AE - Maximum Grade Seen | 1 | 0 | 0 | 0 |
|  | Renal and urinary disorders - Other | 1 | 0 | 0 | 0 |
| Respiratory, thoracic and mediastinal disorders | **Any AE - Maximum Grade Seen | 2 | 0 | 0 | 0 |
|  | Epistaxis | 2 | 0 | 0 | 0 |
|  | Laryngeal inflammation | 1 | 0 | 0 | 0 |
| Skin and subcutaneous tissue disorders | **Any AE - Maximum Grade Seen | 3 | 0 | 0 | 0 |
|  | Alopecia | 1 | 0 | 0 | 0 |
|  | Pruritus | 1 | 0 | 0 | 0 |
|  | Rash acneiform | 1 | 0 | 0 | 0 |
|  | Rash maculo-papular | 1 | 0 | 0 | 0 |
| Vascular disorders | **Any AE - Maximum Grade Seen | 1 | 0 | 0 | 0 |
|  | Hypotension | 1 | 0 | 0 | 0 |
| **** ANY AE - Maximum Grade Seen (Total)** |  | 3 | 9 | 4 | 1 |

**Table S7.2: Attribution to Study Treatment 2 (Oxaliplatin or Paclitaxel)**

***Type and Degree for Non-Severe Adverse Event***

***(Total N Treated=22)***

| **Adverse Event** | | **Grade** | | | |
| --- | --- | --- | --- | --- | --- |
|  |  | 1 | 2 | 3 | 4 |
| **System Organ Class** | **Preferred Term** | 1 | 1 | 0 | 0 |
| Blood and lymphatic system disorders | **Any AE - Maximum Grade Seen |  |  |  |  |
|  | Anemia | 1 | 1 | 0 | 0 |
|  | Blood and lymphatic system disorders - Other | 1 | 0 | 0 | 0 |
| Cardiac disorders | **Any AE - Maximum Grade Seen | 2 | 0 | 0 | 0 |
|  | Sinus tachycardia | 2 | 0 | 0 | 0 |
| Gastrointestinal disorders | **Any AE - Maximum Grade Seen | 8 | 7 | 1 | 0 |
|  | Abdominal pain | 1 | 1 | 1 | 0 |
|  | Bloating | 1 | 0 | 0 | 0 |
|  | Constipation | 9 | 2 | 0 | 0 |
|  | Diarrhea | 7 | 2 | 0 | 0 |
|  | Dry mouth | 1 | 0 | 0 | 0 |
|  | Dyspepsia | 0 | 2 | 0 | 0 |
|  | Esophagitis | 1 | 0 | 0 | 0 |
|  | Gastritis | 0 | 1 | 0 | 0 |
|  | Mucositis oral | 3 | 0 | 0 | 0 |
|  | Nausea | 7 | 6 | 0 | 0 |
|  | Oral dysesthesia | 0 | 2 | 0 | 0 |
|  | Oral hemorrhage | 1 | 0 | 0 | 0 |
|  | Vomiting | 4 | 4 | 0 | 0 |
| General disorders and administration site conditions | **Any AE - Maximum Grade Seen | 7 | 6 | 0 | 0 |
|  | Fatigue | 8 | 5 | 0 | 0 |
|  | Fever | 0 | 1 | 0 | 0 |
|  | Flu like symptoms | 1 | 0 | 0 | 0 |
|  | Malaise | 1 | 0 | 0 | 0 |
|  | Pain | 1 | 0 | 0 | 0 |
| Injury, poisoning and procedural complications | **Any AE - Maximum Grade Seen | 0 | 4 | 0 | 0 |
|  | Infusion related reaction | 0 | 4 | 0 | 0 |
| Investigations | **Any AE - Maximum Grade Seen | 1 | 2 | 3 | 1 |
|  | Alanine aminotransferase increased | 1 | 0 | 0 | 0 |
|  | Aspartate aminotransferase increased | 1 | 0 | 0 | 0 |
|  | Lymphocyte count decreased | 0 | 0 | 2 | 0 |
|  | Neutrophil count decreased | 0 | 2 | 2 | 1 |
|  | Platelet count decreased | 1 | 2 | 0 | 0 |
|  | Weight loss | 1 | 0 | 0 | 0 |
|  | White blood cell decreased | 0 | 1 | 1 | 0 |
| Metabolism and nutrition disorders | **Any AE - Maximum Grade Seen | 4 | 2 | 0 | 0 |
|  | Anorexia | 5 | 1 | 0 | 0 |
|  | Dehydration | 0 | 1 | 0 | 0 |
| Musculoskeletal and connective tissue disorders | **Any AE - Maximum Grade Seen | 1 | 0 | 0 | 0 |
|  | Back pain | 1 | 0 | 0 | 0 |
| Nervous system disorders | **Any AE - Maximum Grade Seen | 8 | 4 | 1 | 0 |
|  | Dizziness | 1 | 0 | 0 | 0 |
|  | Dysesthesia | 5 | 0 | 0 | 0 |
|  | Dysgeusia | 5 | 0 | 0 | 0 |
|  | Headache | 1 | 1 | 0 | 0 |
|  | Paresthesia | 1 | 0 | 0 | 0 |
|  | Peripheral motor neuropathy | 1 | 0 | 0 | 0 |
|  | Peripheral sensory neuropathy | 3 | 4 | 0 | 0 |
|  | Syncope | 0 | 0 | 1 | 0 |
| Psychiatric disorders | **Any AE - Maximum Grade Seen | 1 | 2 | 0 | 0 |
|  | Insomnia | 1 | 2 | 0 | 0 |
| Renal and urinary disorders | **Any AE - Maximum Grade Seen | 1 | 0 | 0 | 0 |
|  | Renal and urinary disorders - Other | 1 | 0 | 0 | 0 |
| Respiratory, thoracic and mediastinal disorders | **Any AE - Maximum Grade Seen | 4 | 0 | 0 | 0 |
|  | Epistaxis | 2 | 0 | 0 | 0 |
|  | Hiccups | 1 | 0 | 0 | 0 |
|  | Hoarseness | 1 | 0 | 0 | 0 |
|  | Laryngeal inflammation | 1 | 0 | 0 | 0 |
| Skin and subcutaneous tissue disorders | **Any AE - Maximum Grade Seen | 3 | 0 | 0 | 0 |
|  | Alopecia | 1 | 0 | 0 | 0 |
|  | Hyperhidrosis | 1 | 0 | 0 | 0 |
|  | Pruritus | 1 | 0 | 0 | 0 |
|  | Rash acneiform | 1 | 0 | 0 | 0 |
|  | Rash maculo-papular | 1 | 0 | 0 | 0 |
| Vascular disorders | **Any AE - Maximum Grade Seen | 2 | 0 | 0 | 0 |
|  | Hot flashes | 1 | 0 | 0 | 0 |
|  | Hypotension | 1 | 0 | 0 | 0 |
| **** ANY AE - Maximum Grade Seen (Total)** |  | 3 | 9 | 4 | 1 |

**Table S7.3: Attribution to Study Treatment 3 (Surgery)**

***Type and Degree for Non-Severe Adverse Event***

***(Total N Treated=22)***

| **Adverse Event** | | **Grade** | | |
| --- | --- | --- | --- | --- |
|  |  | 1 | 2 | 3 |
| **System Organ Class** | **Preferred Term** | 1 | 0 | 0 |
| Cardiac disorders | **Any AE - Maximum Grade Seen |  |  |  |
|  | Sinus tachycardia | 1 | 0 | 0 |
| Gastrointestinal disorders | **Any AE - Maximum Grade Seen | 3 | 0 | 0 |
|  | Abdominal distension | 1 | 0 | 0 |
|  | Bloating | 1 | 0 | 0 |
|  | Constipation | 2 | 0 | 0 |
|  | Flatulence | 2 | 0 | 0 |
| General disorders and administration site conditions | **Any AE - Maximum Grade Seen | 3 | 2 | 0 |
|  | Edema limbs | 1 | 0 | 0 |
|  | Fatigue | 1 | 0 | 0 |
|  | Fever | 2 | 0 | 0 |
|  | Non-cardiac chest pain | 1 | 0 | 0 |
|  | Pain | 1 | 2 | 0 |
| Infections and infestations | **Any AE - Maximum Grade Seen | 0 | 0 | 1 |
|  | Mediastinal infection | 0 | 0 | 1 |
| Injury, poisoning and procedural complications | **Any AE - Maximum Grade Seen | 0 | 1 | 0 |
|  | Wound complication | 1 | 0 | 0 |
|  | Wound dehiscence | 0 | 1 | 0 |
| Investigations | **Any AE - Maximum Grade Seen | 0 | 1 | 0 |
|  | White blood cell decreased | 0 | 1 | 0 |
| Metabolism and nutrition disorders | **Any AE - Maximum Grade Seen | 0 | 1 | 0 |
|  | Hyperphosphatemia | 0 | 1 | 0 |
|  | Hypomagnesemia | 1 | 0 | 0 |
|  | Hyponatremia | 1 | 0 | 0 |
| Musculoskeletal and connective tissue disorders | **Any AE - Maximum Grade Seen | 2 | 1 | 0 |
|  | Arthralgia | 1 | 0 | 0 |
|  | Back pain | 1 | 1 | 0 |
| Psychiatric disorders | **Any AE - Maximum Grade Seen | 2 | 0 | 0 |
|  | Anxiety | 1 | 0 | 0 |
|  | Insomnia | 1 | 0 | 0 |
| Respiratory, thoracic and mediastinal disorders | **Any AE - Maximum Grade Seen | 1 | 1 | 0 |
|  | Pleural effusion | 0 | 1 | 0 |
|  | Respiratory, thoracic and mediastinal disorders - Other | 1 | 1 | 0 |
| **** ANY AE - Maximum Grade Seen (Total)** |  | 2 | 4 | 1 |

**Table S7.4: Any Adverse Event (Related or Unrelated to Treatment)**

***Type and Degree for Non-Severe Adverse Event***

***(Total N Treated=22)***

| **Adverse Event** | | **Grade** | | | |
| --- | --- | --- | --- | --- | --- |
|  |  | 1 | 2 | 3 | 4 |
| **System Organ Class** | **Preferred Term** | 2 | 2 | 0 | 0 |
| Blood and lymphatic system disorders | **Any AE - Maximum Grade Seen |  |  |  |  |
|  | Anemia | 2 | 1 | 0 | 0 |
|  | Blood and lymphatic system disorders - Other | 1 | 1 | 0 | 0 |
| Cardiac disorders | **Any AE - Maximum Grade Seen | 3 | 0 | 0 | 0 |
|  | Sinus tachycardia | 3 | 0 | 0 | 0 |
| Eye disorders | **Any AE - Maximum Grade Seen | 2 | 0 | 0 | 0 |
|  | Blurred vision | 2 | 0 | 0 | 0 |
| Gastrointestinal disorders | **Any AE - Maximum Grade Seen | 10 | 7 | 2 | 0 |
|  | Abdominal distension | 1 | 0 | 0 | 0 |
|  | Abdominal pain | 1 | 1 | 1 | 0 |
|  | Bloating | 2 | 0 | 0 | 0 |
|  | Constipation | 10 | 2 | 0 | 0 |
|  | Diarrhea | 9 | 2 | 0 | 0 |
|  | Dry mouth | 1 | 0 | 0 | 0 |
|  | Dyspepsia | 0 | 2 | 0 | 0 |
|  | Dysphagia | 2 | 1 | 0 | 0 |
|  | Esophagitis | 2 | 0 | 0 | 0 |
|  | Flatulence | 2 | 0 | 0 | 0 |
|  | Gastric ulcer | 0 | 1 | 0 | 0 |
|  | Gastritis | 0 | 1 | 0 | 0 |
|  | Gastroesophageal reflux disease | 1 | 0 | 0 | 0 |
|  | Gastrointestinal disorders - Other | 0 | 0 | 1 | 0 |
|  | Hemorrhoidal hemorrhage | 1 | 0 | 0 | 0 |
|  | Mucositis oral | 3 | 0 | 0 | 0 |
|  | Nausea | 10 | 6 | 0 | 0 |
|  | Oral dysesthesia | 0 | 2 | 0 | 0 |
|  | Oral hemorrhage | 1 | 0 | 0 | 0 |
|  | Stomach pain | 0 | 1 | 0 | 0 |
|  | Vomiting | 6 | 5 | 0 | 0 |
| General disorders and administration site conditions | **Any AE - Maximum Grade Seen | 6 | 9 | 0 | 0 |
|  | Edema limbs | 1 | 1 | 0 | 0 |
|  | Fatigue | 10 | 5 | 0 | 0 |
|  | Fever | 2 | 1 | 0 | 0 |
|  | Flu like symptoms | 1 | 0 | 0 | 0 |
|  | Malaise | 1 | 0 | 0 | 0 |
|  | Non-cardiac chest pain | 1 | 0 | 0 | 0 |
|  | Pain | 2 | 2 | 0 | 0 |
| Infections and infestations | **Any AE - Maximum Grade Seen | 0 | 1 | 1 | 0 |
|  | Mediastinal infection | 0 | 0 | 1 | 0 |
|  | Upper respiratory infection | 0 | 1 | 0 | 0 |
| Injury, poisoning and procedural complications | **Any AE - Maximum Grade Seen | 0 | 5 | 0 | 0 |
|  | Infusion related reaction | 0 | 4 | 0 | 0 |
|  | Wound complication | 1 | 0 | 0 | 0 |
|  | Wound dehiscence | 0 | 1 | 0 | 0 |
| Investigations | **Any AE - Maximum Grade Seen | 1 | 4 | 4 | 1 |
|  | Alanine aminotransferase increased | 1 | 0 | 0 | 0 |
|  | Aspartate aminotransferase increased | 1 | 0 | 0 | 0 |
|  | Lymphocyte count decreased | 0 | 0 | 2 | 0 |
|  | Neutrophil count decreased | 0 | 4 | 2 | 1 |
|  | Platelet count decreased | 1 | 2 | 1 | 0 |
|  | Weight loss | 1 | 0 | 0 | 0 |
|  | White blood cell decreased | 0 | 1 | 1 | 0 |
| Metabolism and nutrition disorders | **Any AE - Maximum Grade Seen | 4 | 5 | 0 | 0 |
|  | Anorexia | 5 | 3 | 0 | 0 |
|  | Dehydration | 0 | 2 | 0 | 0 |
|  | Hyperphosphatemia | 0 | 1 | 0 | 0 |
|  | Hypomagnesemia | 1 | 0 | 0 | 0 |
|  | Hyponatremia | 3 | 0 | 0 | 0 |
| Musculoskeletal and connective tissue disorders | **Any AE - Maximum Grade Seen | 4 | 2 | 0 | 0 |
|  | Arthralgia | 2 | 0 | 0 | 0 |
|  | Back pain | 2 | 1 | 0 | 0 |
|  | Pain in extremity | 0 | 1 | 0 | 0 |
|  | Rotator cuff injury | 1 | 0 | 0 | 0 |
| Neoplasms benign, malignant and unspecified (incl cysts and polyps) | **Any AE - Maximum Grade Seen | 0 | 1 | 0 | 0 |
|  | Tumor pain | 0 | 1 | 0 | 0 |
| Nervous system disorders | **Any AE - Maximum Grade Seen | 11 | 4 | 1 | 0 |
|  | Concentration impairment | 0 | 1 | 0 | 0 |
|  | Dizziness | 3 | 0 | 0 | 0 |
|  | Dysesthesia | 8 | 0 | 0 | 0 |
|  | Dysgeusia | 5 | 0 | 0 | 0 |
|  | Headache | 1 | 1 | 0 | 0 |
|  | Paresthesia | 1 | 0 | 0 | 0 |
|  | Peripheral motor neuropathy | 1 | 0 | 0 | 0 |
|  | Peripheral sensory neuropathy | 3 | 4 | 0 | 0 |
|  | Syncope | 0 | 0 | 1 | 0 |
| Psychiatric disorders | **Any AE - Maximum Grade Seen | 3 | 2 | 0 | 0 |
|  | Agitation | 0 | 1 | 0 | 0 |
|  | Anxiety | 3 | 0 | 0 | 0 |
|  | Depression | 0 | 1 | 0 | 0 |
|  | Insomnia | 1 | 2 | 0 | 0 |
| Renal and urinary disorders | **Any AE - Maximum Grade Seen | 2 | 0 | 0 | 0 |
|  | Renal and urinary disorders - Other | 1 | 0 | 0 | 0 |
|  | Urinary frequency | 1 | 0 | 0 | 0 |
| Respiratory, thoracic and mediastinal disorders | **Any AE - Maximum Grade Seen | 6 | 2 | 0 | 0 |
|  | Cough | 2 | 0 | 0 | 0 |
|  | Dyspnea | 1 | 1 | 0 | 0 |
|  | Epistaxis | 3 | 0 | 0 | 0 |
|  | Hiccups | 1 | 0 | 0 | 0 |
|  | Hoarseness | 1 | 0 | 0 | 0 |
|  | Laryngeal inflammation | 1 | 0 | 0 | 0 |
|  | Pleural effusion | 0 | 1 | 0 | 0 |
|  | Respiratory, thoracic and mediastinal disorders - Other | 1 | 1 | 0 | 0 |
|  | Rhinorrhea | 2 | 0 | 0 | 0 |
| Skin and subcutaneous tissue disorders | **Any AE - Maximum Grade Seen | 3 | 0 | 0 | 0 |
|  | Alopecia | 1 | 0 | 0 | 0 |
|  | Hyperhidrosis | 1 | 0 | 0 | 0 |
|  | Pruritus | 1 | 0 | 0 | 0 |
|  | Rash acneiform | 1 | 0 | 0 | 0 |
|  | Rash maculo-papular | 1 | 0 | 0 | 0 |
|  | Skin and subcutaneous tissue disorders - Other | 1 | 0 | 0 | 0 |
| Vascular disorders | **Any AE - Maximum Grade Seen | 3 | 1 | 0 | 0 |
|  | Hot flashes | 2 | 0 | 0 | 0 |
|  | Hypotension | 1 | 0 | 0 | 0 |
|  | Vascular disorders - Other | 0 | 1 | 0 | 0 |
| **** ANY AE - Maximum Grade Seen (Total)** |  | 2 | 12 | 5 | 1 |

**Table S7.5: Adverse Events Attributable to Any Treatment**

***Type and Degree for Non-Severe Adverse Event***

***(Total N Treated=22)***

| **Adverse Event** | | **Grade** | | | |
| --- | --- | --- | --- | --- | --- |
|  |  | 1 | 2 | 3 | 4 |
| **System Organ Class** | **Preferred Term** | 1 | 1 | 0 | 0 |
| Blood and lymphatic system disorders | **Any AE - Maximum Grade Seen |  |  |  |  |
|  | Anemia | 1 | 1 | 0 | 0 |
|  | Blood and lymphatic system disorders - Other | 1 | 0 | 0 | 0 |
| Cardiac disorders | **Any AE - Maximum Grade Seen | 3 | 0 | 0 | 0 |
|  | Sinus tachycardia | 3 | 0 | 0 | 0 |
| Gastrointestinal disorders | **Any AE - Maximum Grade Seen | 8 | 7 | 1 | 0 |
|  | Abdominal distension | 1 | 0 | 0 | 0 |
|  | Abdominal pain | 1 | 1 | 1 | 0 |
|  | Bloating | 2 | 0 | 0 | 0 |
|  | Constipation | 9 | 2 | 0 | 0 |
|  | Diarrhea | 7 | 2 | 0 | 0 |
|  | Dry mouth | 1 | 0 | 0 | 0 |
|  | Dyspepsia | 0 | 2 | 0 | 0 |
|  | Esophagitis | 1 | 0 | 0 | 0 |
|  | Flatulence | 2 | 0 | 0 | 0 |
|  | Gastritis | 0 | 1 | 0 | 0 |
|  | Mucositis oral | 3 | 0 | 0 | 0 |
|  | Nausea | 7 | 6 | 0 | 0 |
|  | Oral dysesthesia | 0 | 2 | 0 | 0 |
|  | Oral hemorrhage | 1 | 0 | 0 | 0 |
|  | Vomiting | 4 | 4 | 0 | 0 |
| General disorders and administration site conditions | **Any AE - Maximum Grade Seen | 5 | 8 | 0 | 0 |
|  | Edema limbs | 1 | 0 | 0 | 0 |
|  | Fatigue | 8 | 5 | 0 | 0 |
|  | Fever | 2 | 1 | 0 | 0 |
|  | Flu like symptoms | 1 | 0 | 0 | 0 |
|  | Malaise | 1 | 0 | 0 | 0 |
|  | Non-cardiac chest pain | 1 | 0 | 0 | 0 |
|  | Pain | 2 | 2 | 0 | 0 |
| Infections and infestations | **Any AE - Maximum Grade Seen | 0 | 0 | 1 | 0 |
|  | Mediastinal infection | 0 | 0 | 1 | 0 |
| Injury, poisoning and procedural complications | **Any AE - Maximum Grade Seen | 0 | 5 | 0 | 0 |
|  | Infusion related reaction | 0 | 4 | 0 | 0 |
|  | Wound complication | 1 | 0 | 0 | 0 |
|  | Wound dehiscence | 0 | 1 | 0 | 0 |
| Investigations | **Any AE - Maximum Grade Seen | 1 | 2 | 3 | 1 |
|  | Alanine aminotransferase increased | 1 | 0 | 0 | 0 |
|  | Aspartate aminotransferase increased | 1 | 0 | 0 | 0 |
|  | Lymphocyte count decreased | 0 | 0 | 2 | 0 |
|  | Neutrophil count decreased | 0 | 2 | 2 | 1 |
|  | Platelet count decreased | 1 | 2 | 0 | 0 |
|  | Weight loss | 1 | 0 | 0 | 0 |
|  | White blood cell decreased | 0 | 1 | 1 | 0 |
| Metabolism and nutrition disorders | **Any AE - Maximum Grade Seen | 4 | 3 | 0 | 0 |
|  | Anorexia | 5 | 1 | 0 | 0 |
|  | Dehydration | 0 | 1 | 0 | 0 |
|  | Hyperphosphatemia | 0 | 1 | 0 | 0 |
|  | Hypomagnesemia | 1 | 0 | 0 | 0 |
|  | Hyponatremia | 1 | 0 | 0 | 0 |
| Musculoskeletal and connective tissue disorders | **Any AE - Maximum Grade Seen | 2 | 1 | 0 | 0 |
|  | Arthralgia | 1 | 0 | 0 | 0 |
|  | Back pain | 2 | 1 | 0 | 0 |
| Nervous system disorders | **Any AE - Maximum Grade Seen | 8 | 4 | 1 | 0 |
|  | Dizziness | 1 | 0 | 0 | 0 |
|  | Dysesthesia | 5 | 0 | 0 | 0 |
|  | Dysgeusia | 5 | 0 | 0 | 0 |
|  | Headache | 1 | 1 | 0 | 0 |
|  | Paresthesia | 1 | 0 | 0 | 0 |
|  | Peripheral motor neuropathy | 1 | 0 | 0 | 0 |
|  | Peripheral sensory neuropathy | 3 | 4 | 0 | 0 |
|  | Syncope | 0 | 0 | 1 | 0 |
| Psychiatric disorders | **Any AE - Maximum Grade Seen | 2 | 2 | 0 | 0 |
|  | Anxiety | 1 | 0 | 0 | 0 |
|  | Insomnia | 1 | 2 | 0 | 0 |
| Renal and urinary disorders | **Any AE - Maximum Grade Seen | 1 | 0 | 0 | 0 |
|  | Renal and urinary disorders - Other | 1 | 0 | 0 | 0 |
| Respiratory, thoracic and mediastinal disorders | **Any AE - Maximum Grade Seen | 4 | 1 | 0 | 0 |
|  | Epistaxis | 2 | 0 | 0 | 0 |
|  | Hiccups | 1 | 0 | 0 | 0 |
|  | Hoarseness | 1 | 0 | 0 | 0 |
|  | Laryngeal inflammation | 1 | 0 | 0 | 0 |
|  | Pleural effusion | 0 | 1 | 0 | 0 |
|  | Respiratory, thoracic and mediastinal disorders - Other | 1 | 1 | 0 | 0 |
| Skin and subcutaneous tissue disorders | **Any AE - Maximum Grade Seen | 3 | 0 | 0 | 0 |
|  | Alopecia | 1 | 0 | 0 | 0 |
|  | Hyperhidrosis | 1 | 0 | 0 | 0 |
|  | Pruritus | 1 | 0 | 0 | 0 |
|  | Rash acneiform | 1 | 0 | 0 | 0 |
|  | Rash maculo-papular | 1 | 0 | 0 | 0 |
| Vascular disorders | **Any AE - Maximum Grade Seen | 2 | 0 | 0 | 0 |
|  | Hot flashes | 1 | 0 | 0 | 0 |
|  | Hypotension | 1 | 0 | 0 | 0 |
| **** ANY AE - Maximum Grade Seen (Total)** |  | 2 | 10 | 4 | 1 |

**Table S7.6: Any Adverse Event (Related or Unrelated to Treatment) with a Frequency > 20%**

***Type and Degree for Non-Severe Adverse Event***

***(Total N Treated=22)***

| **Adverse Event Summary** | | **Grade** | | | |
| --- | --- | --- | --- | --- | --- |
|  |  | 1 | 2 | 3 | 4 |
| **System Organ Class** | **Preferred Term** |  |  |  |  |
| Gastrointestinal disorders | Constipation | 10 | 2 | 0 | 0 |
|  | Diarrhea | 9 | 2 | 0 | 0 |
|  | Nausea | 10 | 6 | 0 | 0 |
|  | Vomiting | 6 | 5 | 0 | 0 |
| General disorders and administration site conditions | Fatigue | 10 | 5 | 0 | 0 |
| Investigations | Neutrophil count decreased | 0 | 4 | 2 | 1 |
| Metabolism and nutrition disorders | Anorexia | 5 | 3 | 0 | 0 |
| Nervous system disorders | Dysesthesia | 8 | 0 | 0 | 0 |
|  | Dysgeusia | 5 | 0 | 0 | 0 |
|  | Peripheral sensory neuropathy | 3 | 4 | 0 | 0 |

**Table S7.7: Grade 3 or Higher Adverse Events (Related or Unrelated to Treatment)**

***Type and Degree for Non-Severe Adverse Event (Grade 3 or Higher)***

***(Total N Treated=22)***

***(Total Patients with Grade 3 or Higher Adverse Events = 9)***

| **Adverse Event** | | **Grade** | |
| --- | --- | --- | --- |
|  |  | 3 | 4 |
| **System Organ Class** | **Preferred Term** | 2 | 0 |
| Gastrointestinal disorders | **Any AE - Maximum Grade Seen |  |  |
|  | Abdominal pain | 1 | 0 |
|  | Gastrointestinal disorders - Other | 1 | 0 |
| Infections and infestations | **Any AE - Maximum Grade Seen | 1 | 0 |
|  | Mediastinal infection | 1 | 0 |
| Investigations | **Any AE - Maximum Grade Seen | 4 | 1 |
|  | Lymphocyte count decreased | 2 | 0 |
|  | Neutrophil count decreased | 2 | 1 |
|  | Platelet count decreased | 1 | 0 |
|  | White blood cell decreased | 1 | 0 |
| Nervous system disorders | **Any AE - Maximum Grade Seen | 1 | 0 |
|  | Syncope | 1 | 0 |
| **** ANY AE - Maximum Grade Seen (Total)** |  | 5 | 1 |

**Table S7.8: Grade 3 or Higher Adverse Events Attributable to Treatment (Any)**

***Type and Degree for Grade 3 or Higher AEs Attributable to Treatment (ANY)***

***(Total N Treated=22)***

***(Total Patients with Grade 3 or Higher Adverse Events = 9)***

| **Adverse Event** | | **Grade** | |
| --- | --- | --- | --- |
|  |  | 3 | 4 |
| **System Organ Class** | **Preferred Term** | 1 | 0 |
| Gastrointestinal disorders | **Any AE - Maximum Grade Seen |  |  |
|  | Abdominal pain | 1 | 0 |
| Infections and infestations | **Any AE - Maximum Grade Seen | 1 | 0 |
|  | Mediastinal infection | 1 | 0 |
| Investigations | **Any AE - Maximum Grade Seen | 3 | 1 |
|  | Lymphocyte count decreased | 2 | 0 |
|  | Neutrophil count decreased | 2 | 1 |
|  | White blood cell decreased | 1 | 0 |
| Nervous system disorders | **Any AE - Maximum Grade Seen | 1 | 0 |
|  | Syncope | 1 | 0 |
| **** ANY AE - Maximum Grade Seen (Total)** |  | 4 | 1 |

**Abbreviations for Table S6:** AE, adverse event.

**Table S8. Clinical data of patients with recurrence after surgery**

Among 14 patients undergoing surgery, seven patients had recurrence after surgery. Median days of recurrence after surgery were 120 days (range: 28 to 730 days). Liver (N=3, 42.9%) and brain (N=2, 28.6%) were the two most common sites of recurrence.

| Patient | Time to recurrence post op | Location of recurrence |
| --- | --- | --- |
| 1 | 730 days | Lung |
| 2 | 208 days | Liver |
| 3 | 120 days | Liver, anterior thoracic lymph nodes |
| 4 | 620 days | Femur neck |
| 5 | 28 days | Brain |
| 6 | 122 days | Brain |
| 7 | 73 days | Liver, peritoneum |

**Table S9. Summary of surgical outcomes and complications in this study and other chemoradiation trials with an institutional reference**

|  | **R0 resection rate** | **Complication rate** |  | **Ref** |
| --- | --- | --- | --- | --- |
|  |  | **Any grade** | **Grade 3-5** |  |
| **Present study** |  |  |  | - |
| All patients (N=22) | 68.2% (N=15/22) | 31.8% (N=7/22) | 4.5% (N=1/22) |  |
| Patients undergoing surgery (N=14)*^1^ | 100% (N=14/14) | 50.0% (N=7/14)  30-day mortality: 0% | 7.1% (N=1/14) |  |
| **Institutional reference*^2^ (N=302)** | 96.7% (N=292/302) | 30-day mortality: 0.7% (N=2/302) | 37.8% (N=114/302)*^3^ | 1 |
| **CROSS trial (N=171 in CRT group)** | 92% (N=148/161) | Pulmonary complication: 46% (N=78/168)  Cardiac complication: 21% (N=36/168)  30-day mortality: 4% (N=6/168) |  | 2 |
| **ESOPEC trial (N=217 in CROSS group)** | 95% (N=171/180) | 62.7% in 180 patients*^3^  30-day mortality: 1.7% in 180 patients | 27.7% in 180 patients*^3^ | 3 |
| **TOPGEAR trial (N=286 in CRT group, N=98 with GEJ tumor)** | 92% (N=202/225)*^4^ | 30-day mortality: 0% (N=0/230) | 18% (N=41/230) | 4 |

***^1^** Surgery in one patient was terminated due to unresectable disease determined during the procedure and is not included in this table.

***^2^** 239 (79.1%) patients received preoperative chemoradiation, then proceed with surgery.

*^3^ Complications were based on the Clavien-Dindo classification system in this study. Other studies used NCI CTCAE for assessing adverse events.

*^4^ Out of 286 patients, 84% (N=241) cases proceed to surgery.

**Abbreviations**: CRT, chemoradiotherapy; GEJ, gastroesophageal junction.

References

1. J Gastrointest Surg. 2024 May;28(5):634-639.

2. N Engl J Med. 2012 May 31;366(22):2074-84.

3. JCO 42, LBA1-LBA1(2024). 10.1200/JCO.2024.42.17_suppl.LBA1

4. N Engl J Med. 2024 Sep 14. doi: 10.1056/NEJMoa2405195.

**Figure S1. Study schema**

**
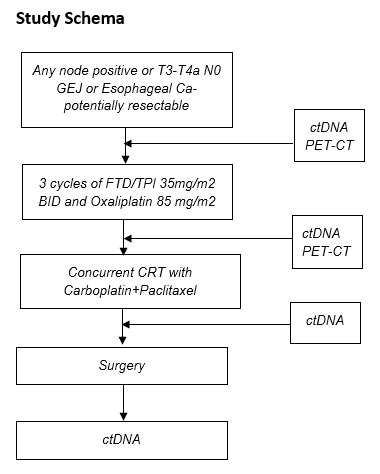
**

**Abbreviations:** BID, twice daily; Ca, carcinoma; CRT, chemoradiotherapy; ctDNA, circulating tumor DNA; FTD/TPI, Trifluridine/Tipiracil; GEJ, gastroesophageal junction; PET-CT, positron emission tomography-computed tomography.

**Figure S2: Summary of PET-CT findings at prespecified treatment timepoints**

**
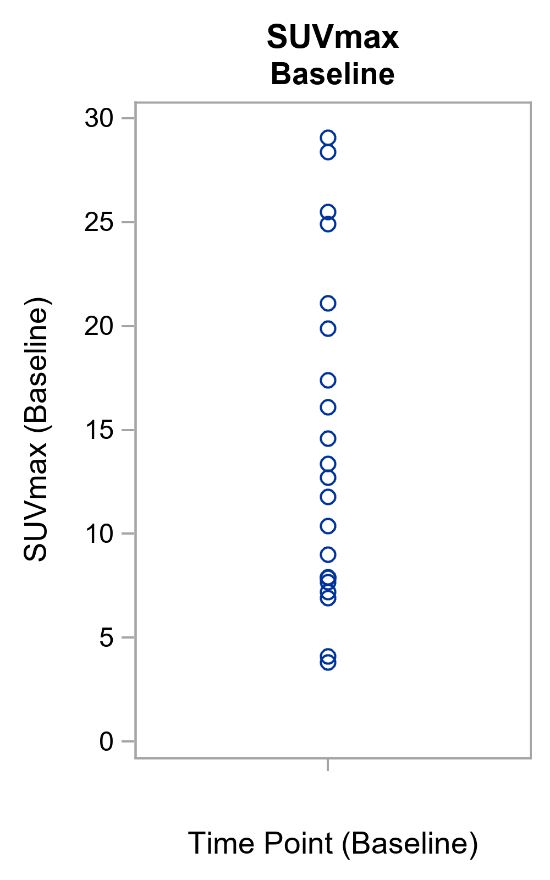
(A) Dot plots – SUVmax by time point including baseline, after induction chemotherapy, and after chemoradiation therapy**

**
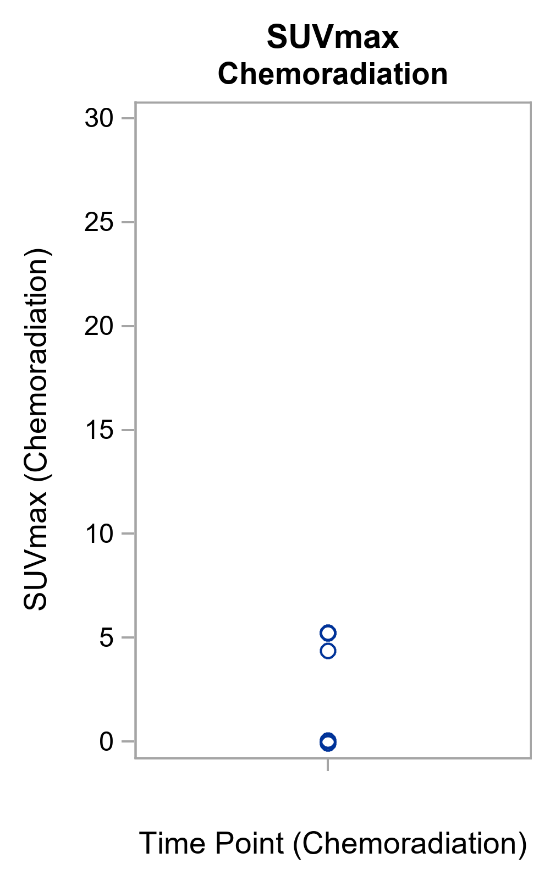

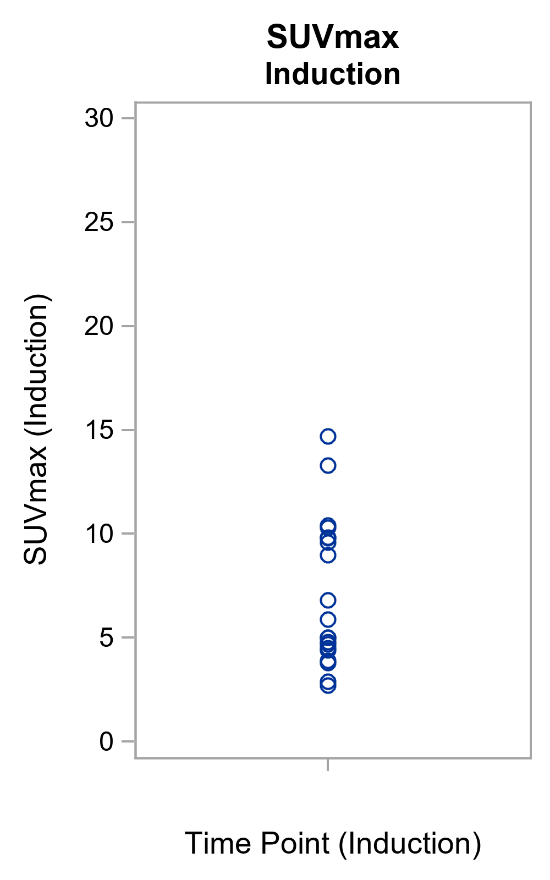
**

**(B) SUV expression changes over the treatment course in each patient**


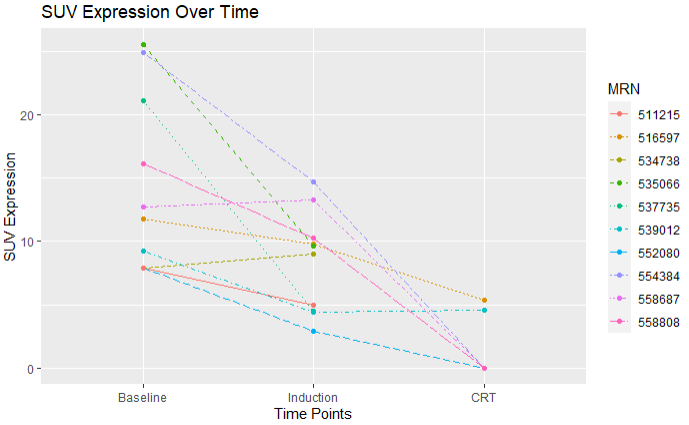


**(C) Changes in mean SUV expression over the treatment course**


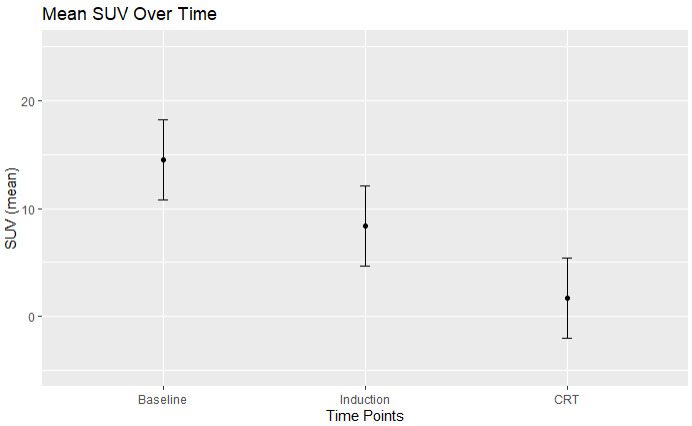


**(D) Number of patients evaluated at each prespecified timepoint**

| **Time Point** | **Sample Size (N)** |
| --- | --- |
| Baseline SUVmax | 22 |
| Induction SUVmax | 20 |
| Chemoradiation SUVmax | 7 |

**(E) Mean and median of SUV at each prespecified timepoint**

|  | **SUV** | |
| --- | --- | --- |
|  | **Mean (SD)** | **Median (IQR)** |
| **Baseline** | 14.5 (7.028) | 12.25 (8.225, 19.85) |
| **Induction** | 8.352 (3.995) | 9.285 (4.625, 10.18) |
| **CRT** | 1.667 (2.594) | 0.0 (0.0, 3.45) |
| **Overall** | 9.174 (7.072) | 8.435 (4.525, 12.48) |

**Abbreviations:** CRT, chemoradiotherapy; IQR, interquartile range; SD, standard deviation; SUV, standardized uptake value.

**Figure S3: (A) Changes in ctDNA expression in each patient over the treatment course (B) Mean ctDNA expression in 10 patients over the treatment course**

**(A) Changes in ctDNA expression in each patient over the treatment course**


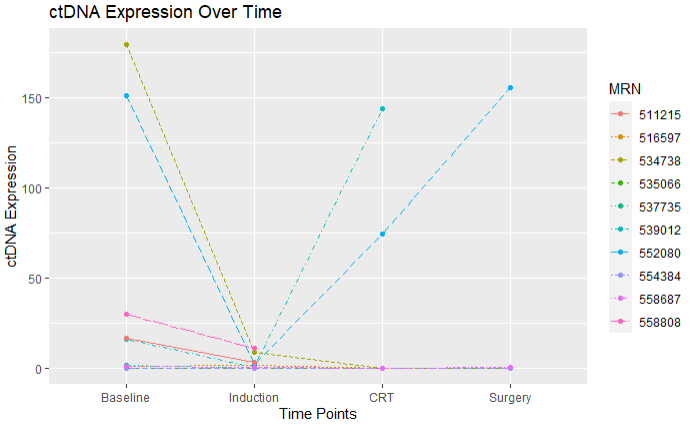


**(B) Mean ctDNA expression in 10 patients over the treatment course**


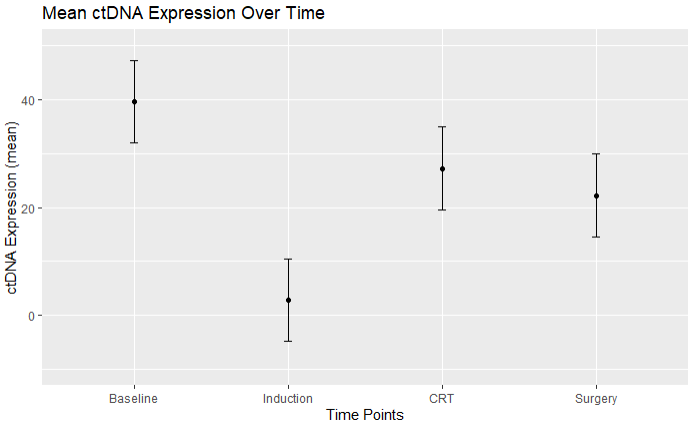


**Abbreviation:** ctDNA, circulating tumor DNA.

**Figure S4: Correlations between SUV and ctDNA expression at prespecified different timepoints**

**(A) At baseline**


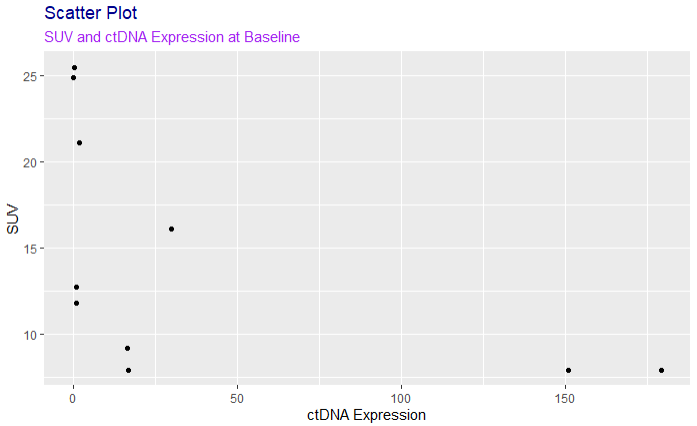


| **rho** | **P-Value** |
| --- | --- |
| -0.7669 | **0.009638** |

**(B) After induction chemotherapy**


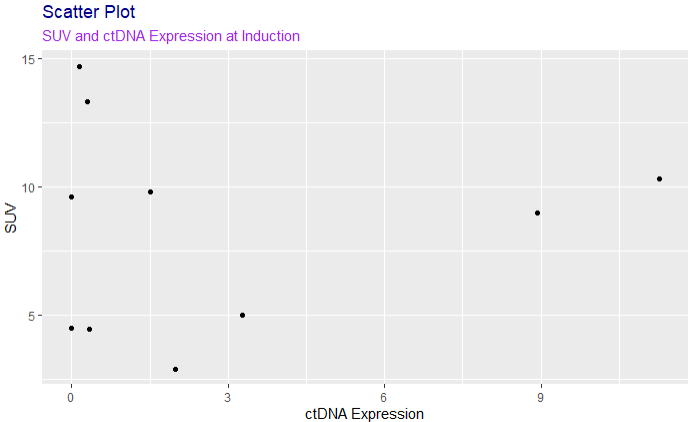


| **rho** | **P-Value** |
| --- | --- |
| -0.08511 | 0.8152 |

**(C) After CRT**


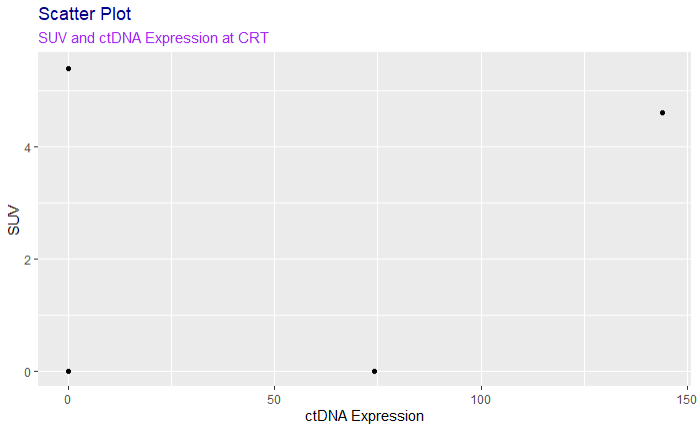


| **rho** | **P-Value** |
| --- | --- |
| 0.125 | 0.8413 |

**Abbreviations:** CRT, chemoradiotherapy; ctDNA, circulating tumor DNA; SUV, standardized uptake value.

**Figure S5: Kaplan-Meier curves of ctDNA clearance based on clinical factors**

**(A) Kaplan-Meier curves of ctDNA clearance based on tumor response score**


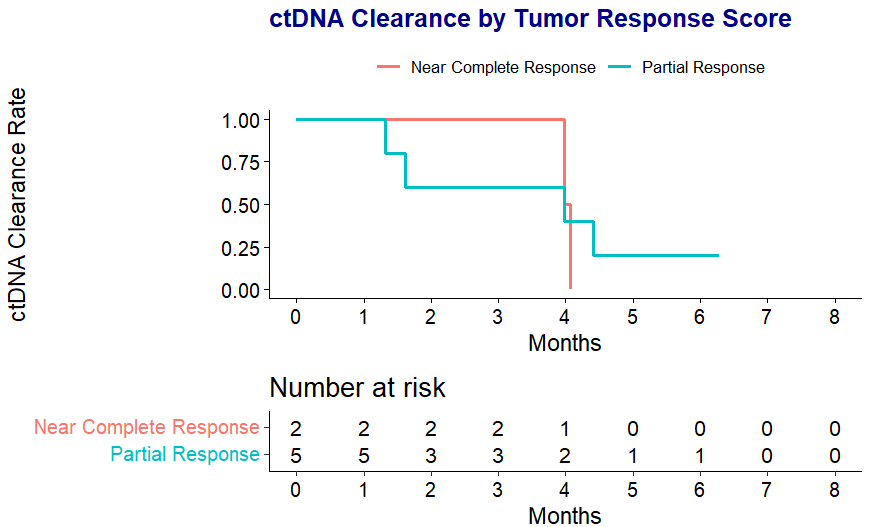


|  | **Median (months)** | **0.95 LCL** | **0.95 UCL** | **P-Value** |
| --- | --- | --- | --- | --- |
| **Near Complete** | 4.025 | 3.975 |  | 0.800 |
| **Partial** | 3.975 | 1.610 |  |  |

**(B)** **Kaplan-Meier curves of ctDNA clearance based on tumor T-stage**


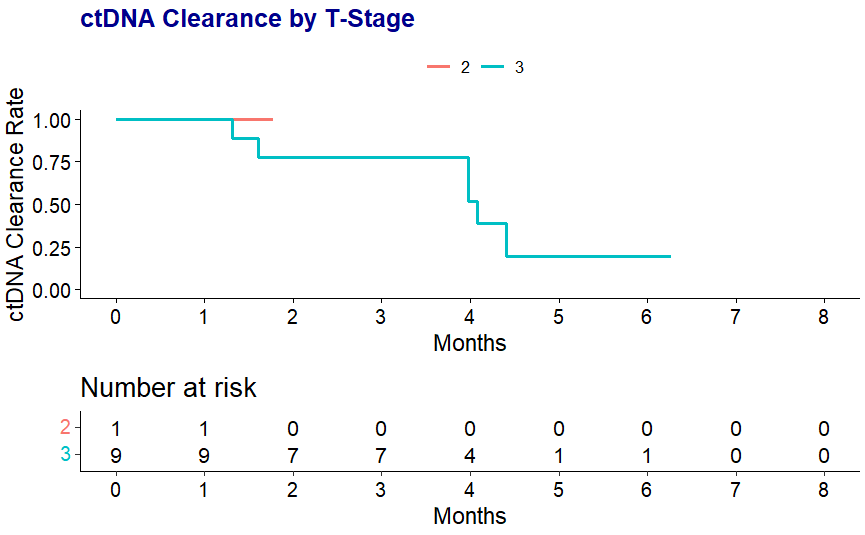


|  | **Median (months)** | **0.95 LCL** | **0.95 UCL** | **P-Value** |
| --- | --- | --- | --- | --- |
| **2** |  |  |  | 0.600 |
| **3** | 4.074 | 3.975 |  |  |

**(C) Kaplan-Meier curves of ctDNA clearance based on tumor N-stage**


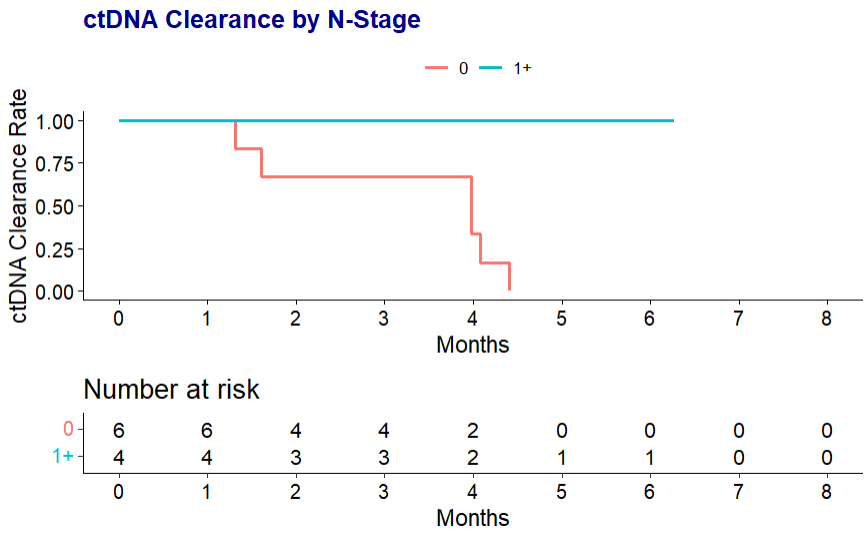


|  | **Median (months)** | **0.95 LCL** | **0.95 UCL** | **P-Value** |
| --- | --- | --- | --- | --- |
| **0** | 3.975 | 1.610 |  | **0.030** |
| **1+** |  |  |  |  |

(D) **Kaplan-Meier curves of ctDNA clearance based on PET-CT response**


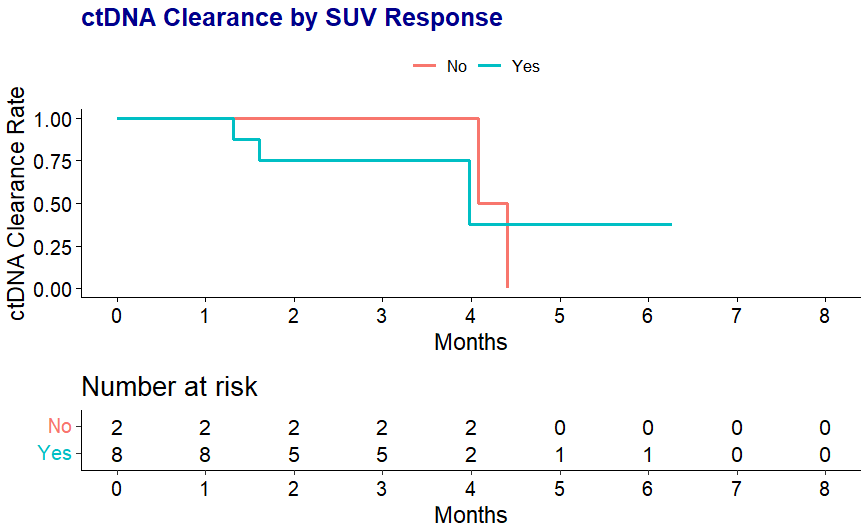


|  | **Median (months)** | **0.95 LCL** | **0.95 UCL** | **P-Value** |
| --- | --- | --- | --- | --- |
| **No** | 4.238 | 4.074 |  | 0.900 |
| **Yes** | 3.975 | 3.975 |  |  |

**Abbreviations:** ctDNA, circulating tumor DNA; PET-CT, positron emission tomography-computed tomography; SUV, standardized uptake value.

**Figure S6: Kaplan-Meier curves of overall survival and progression-free survival based on radiological and pathological response in 10 patients with ctDNA information**

**(A) Overall survival of 10 patients**


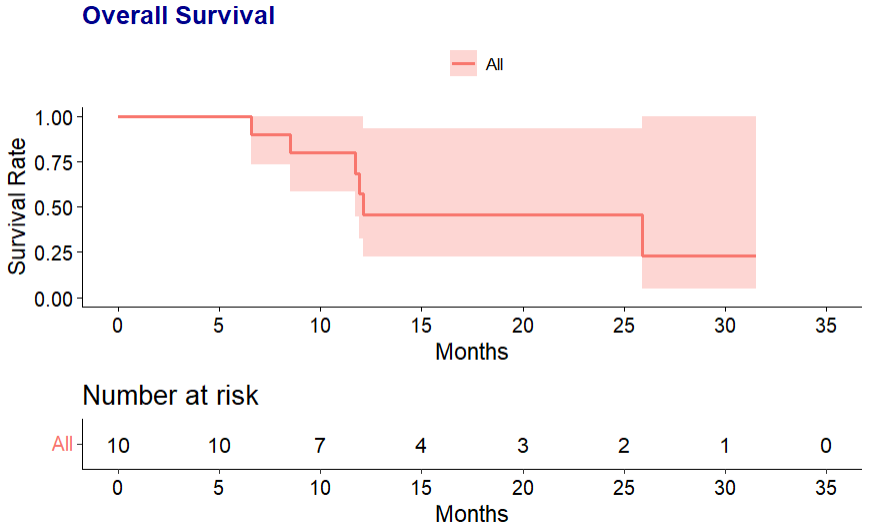


| **Median (months)** | **0.95 LCL** | **0.95 UCL** |
| --- | --- | --- |
| 12.12 | 11.73 |  |

**(B) Progression-free survival of 10 patients**


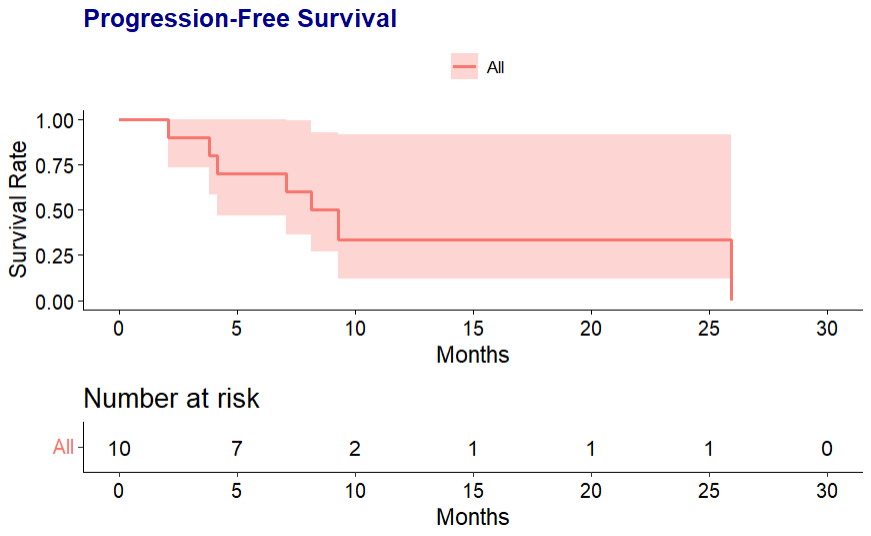


| **Median (months)** | **0.95 LCL** | **0.95 UCL** |
| --- | --- | --- |
| 8.723 | 4.140 |  |

**(C) Overall survival by best response**


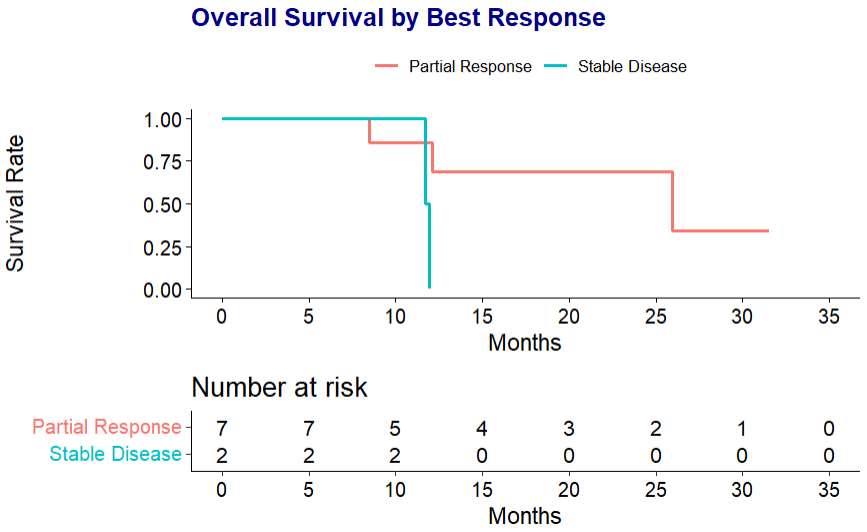


|  | **Median (months)** | **0.95 LCL** | **0.95 UCL** | **P-Value** |
| --- | --- | --- | --- | --- |
| **Partial Response** | 25.92 | 12.12 |  | 0.060 |
| **Stable Disease** | 11.83 | 11.73 |  |  |

**(D) Progression-free survival by best response**


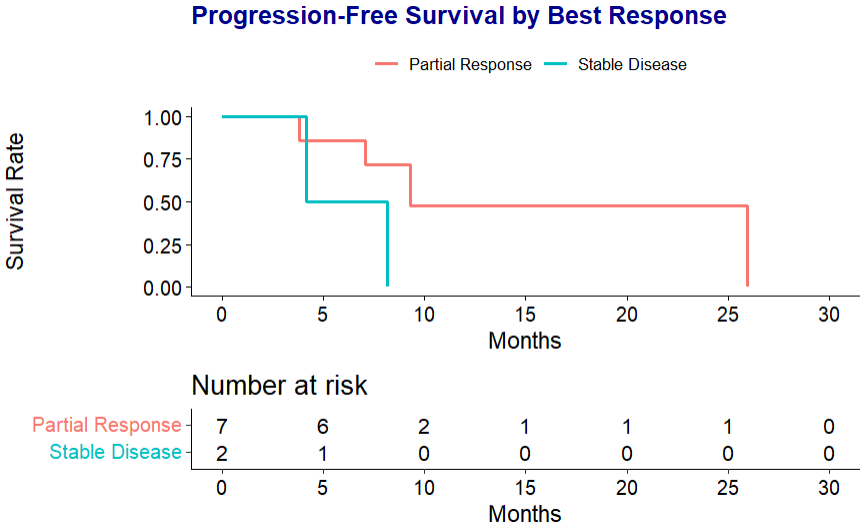


|  | **Median (months)** | **0.95 LCL** | **0.95 UCL** | **P-Value** |
| --- | --- | --- | --- | --- |
| **Partial Response** | 9.298 | 7.097 |  | 0.100 |
| **Stable Disease** | 6.144 | 4.140 |  |  |

**(E) Overall survival by tumor response score**


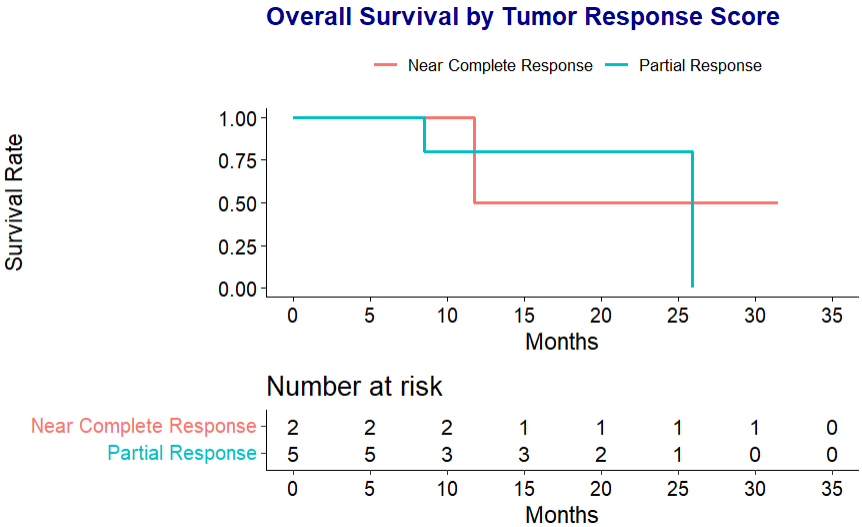


|  | **Median (months)** | **0.95 LCL** | **0.95 UCL** | **P-Value** |
| --- | --- | --- | --- | --- |
| **Partial Response** | 11.73 | 11.73 |  | 0.800 |
| **Stable Disease** | 25.92 |  |  |  |

**(F) Progression-free survival by tumor response score**


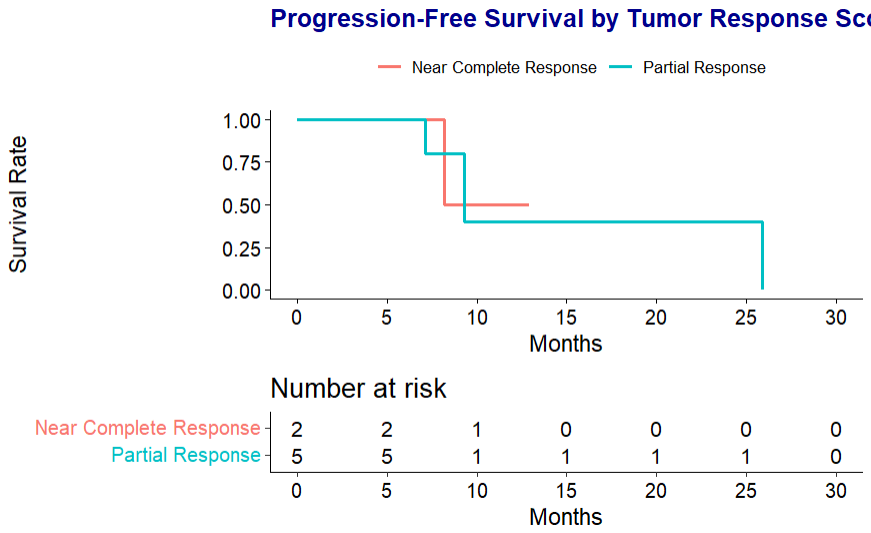


|  | **Median (months)** | **0.95 LCL** | **0.95 UCL** | **P-Value** |
| --- | --- | --- | --- | --- |
| **Partial Response** | 8.148 | 8.148 |  | 1.00 |
| **Stable Disease** | 9.298 | 9.298 |  |  |

**(G) Number of patients for radiological and pathological response outcomes**

|  |  | **N** |
| --- | --- | --- |
|  |  | **10** |
| **Best Response** | Stable Disease | 2 (22.22%) |
|  | Partial Response | 7 (77.77%) |
| **Tumor Regression Score** | Near Complete Response | 2 (28.57%) |
|  | Partial Response | 5 (41.43%) |

**Abbreviation:** ctDNA, circulating tumor DNA.
